# DBT affects sleep in both circadian and non-circadian neurons

**DOI:** 10.1371/journal.pgen.1010035

**Published:** 2022-02-09

**Authors:** Jialin Wang, Jin-Yuan Fan, Zhangwu Zhao, Stephane Dissel, Jeffrey Price

**Affiliations:** 1 Department of Entomology and MOA Key Lab of Pest Monitoring and Green Management, College of Plant Protection, China Agricultural University, Beijing, China; 2 School of Biological and Chemical Sciences, University of Missouri-Kansas City, Kansas City, Missouri, United States of America; Universidad de Valparaiso, CHILE

## Abstract

Sleep is a very important behavior observed in almost all animals. Importantly, sleep is subject to both circadian and homeostatic regulation. The circadian rhythm determines the daily alternation of the sleep-wake cycle, while homeostasis mediates the rise and dissipation of sleep pressure during the wake and sleep period. As an important kinase, *dbt* plays a central role in both circadian rhythms and development. We investigated the sleep patterns of several ethyl methanesulfonate-induced *dbt* mutants and discuss the possible reasons why different sleep phenotypes were shown in these mutants. In order to reduce DBT in all neurons in which it is expressed, CRISPR-Cas9 was used to produce flies that expressed GAL4 in frame with the *dbt* gene at its endogenous locus, and knock-down of DBT with this construct produced elevated sleep during the day and reduced sleep at night. Loss of sleep at night is mediated by *dbt* loss during the sleep/wake cycle in the adult, while the increased sleep during the day is produced by reductions in *dbt* during development and not by reductions in the adult. Additionally, using targeted RNA interference, we uncovered the contribution of *dbt* on sleep in different subsets of neurons in which *dbt* is normally expressed. Reduction of *dbt* in circadian neurons produced less sleep at night, while lower expression of *dbt* in noncircadian neurons produced increased sleep during the day. Importantly, independently of the types of neurons where *dbt* affects sleep, we demonstrate that the PER protein is involved in DBT mediated sleep regulation.

## Author summary

Doubletime (*dbt*) is known as a kinase orthologous to mammalian Casein Kinase I ε (CKIε) and Casein Kinase I δ (CKIδ), which are involved in various biological processes and play an important role in regulation of circadian rhythm. In this study, we first analyzed the role of *dbt* on sleep in *Drosophila*, and then mapped its expression pattern and further neuronal mechanisms, in which DBT importantly regulates sleep through PER in both non-clock neurons and clock neurons.

## Introduction

Sleep is a conserved behavior that has been observed in a variety of species ranging from insects to mammals [1,2]. During sleep, the organism remains quiescent and has a lower response to outside stimulation. Behavioral and electroencephalogram (EEG) studies have suggested that sleep is controlled by circadian and homeostatic processes [3]. The circadian rhythm process could clearly reflect the changing tendency of sleep and wake activity during the day, and it limits sleep amount in a day [4,5]. As for the homeostatic process, it is produced by the accumulation of sleep pressure in the wake period and the release of this pressure in the following sleep period [1]. Although nearly all animals need sleep, the purpose and function of sleep remain poorly understood. With the development of molecular biology, many aspects of life have been addressed at the gene level, including the mechanisms of sleep. Over recent years, many genes have been shown to be involved in sleep regulation [1,6].

As one of the most important clock genes, DBT is known as a kinase orthologous to mammalian kinases CKIε and CKIδ [7,8] and contributes to many different kinds of functions, including circadian rhythm [7–12], planar cell polarity [13,14], programmed cell death [15,16] and growth. Although DBT/CKIε is involved in various biological processes including in the circadian rhythm, its relationship with sleep is still unclear. Intriguingly, a previous report showed that functional consequences of a CKIδ mutation caused familial advanced sleep phase syndrome (FASPS) in mammals [17], suggesting that DBT/CKIε could be involved in sleep regulation. However, it is still not known how DBT/CKIε affects sleep in *Drosophila* and other organisms. In this study, the sleep phenotype and neuronal mechanism by which DBT affects sleep were analyzed. As a strong effect in *dbt* mutants was observed, a *dbt*-GAL4 line was constructed with CRISPR/Cas9 to address the localization of DBT expression, and then we used the GAL4/UAS binary expression approach to drive the expression of *dbt*^RNAi^ in specific neurons in which DBT is expressed, including the Mushroom Bodies (MB), Fan-Shaped Body (FSB), Ellipsoid Body (EB), Pars Intercerebralis (PI) and clock neurons [18]. Results show that DBT affects sleep in both clock neurons and some parts of the central complex and Mushroom Body, and these effects on sleep are based on PER protein.

## Results

### Sleep in EMS *dbt* mutants exhibits altered amounts and phase changes consistent with the circadian period changes

In order to determine if the *dbt* gene affects sleep we analyzed sleep behavior of two different *dbt* mutants-*dbt*^S^ and *dbt*^L^, which produce short period and long period circadian rhythm respectively. The *dbt*^S^ and *dbt*^L^ are two point-mutations of *dbt*, with the point mutation resulting in an amino acid change of Pro47Ser in *dbt*^S^ flies and Met80Ile in *dbt*^L^ flies [7]. These two mutations were reported to affect stability and circadian rhythmicity of PER by altering PER phosphorylation [8]. In the *dbt*^S^ mutant, the PER protein has a more rapid phosphorylation [9], and the circadian period of the *dbt*^S^ mutant in DD is decreased to around 18 hours [8]. By contrast, in the *dbt*^L^ mutant, the PER protein remains longer than wildtype in DD and the circadian period in DD is increased to around 26.8h [8].

**Fig 1:**
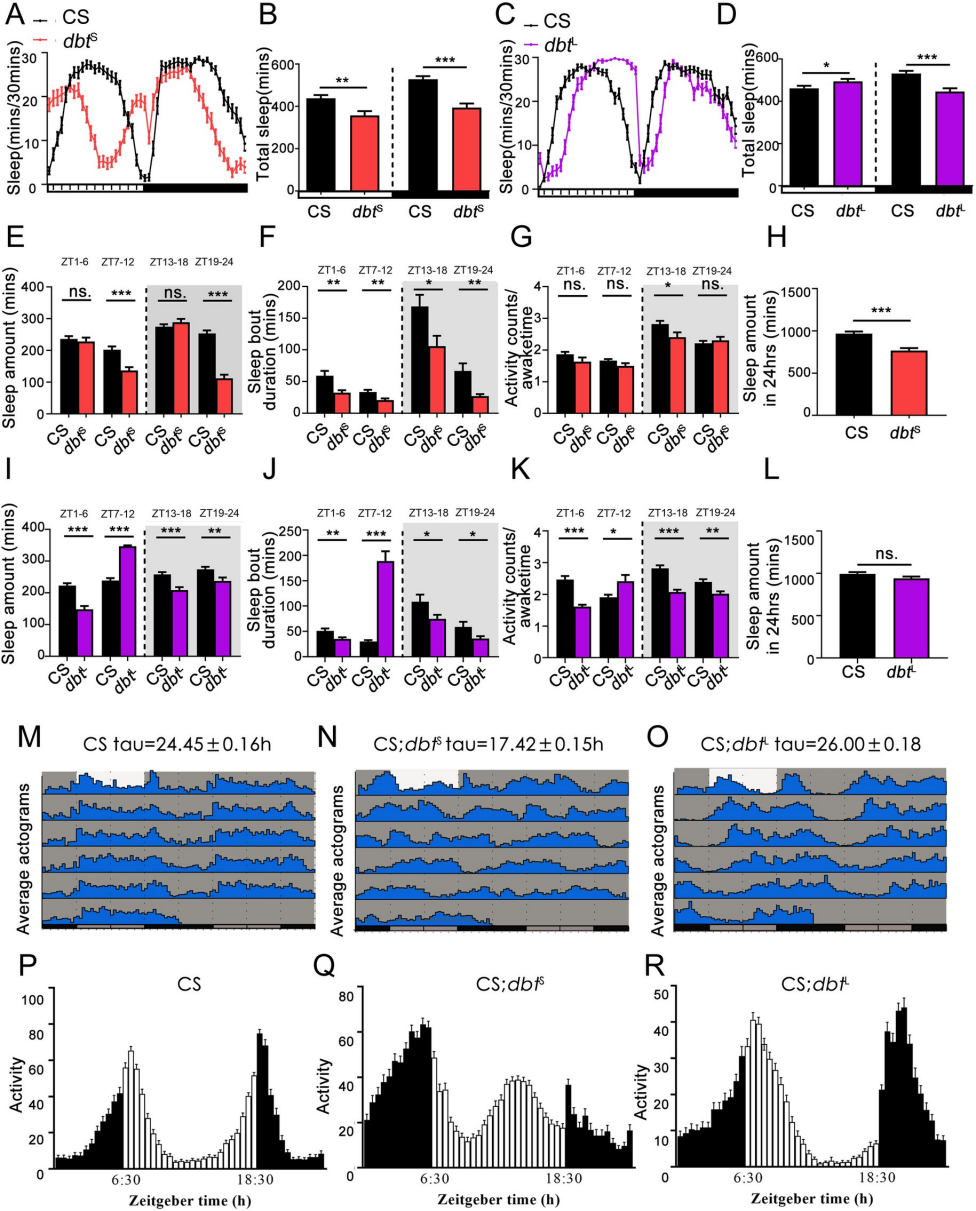
Different period-altering *dbt* mutants cause different sleep phenotypes, but both of them cause a sleep reduction during the nighttime. (A) Sleep profiles of *dbt*^S^ flies. n = 47 in each group. (B) Average sleep amount during the daytime and nighttime in *dbt*^S^ flies (daytime: t = 3.376, df = 92, **p = 0.0011; nighttime: t = 5.776, df = 92, ***p<0.0001 by unpaired t-test). n = 47 in each group. (C) Sleep profiles of *dbt*^L^ flies. n = 47 in each group. (D) Average sleep amount during the daytime and nighttime in *dbt*^L^ flies (daytime: t = 2.025, df = 92, *p = 0.0458; nighttime: t = 4.355, df = 92, ***p <0.0001 by unpaired t-test). n = 47 in each group. (E) Total sleep in *dbt*^S^ flies through a day. The four groups of bars are representing the total sleep of CS (black bar) and *dbt*^S^ mutants (orange bar) in ZT1-6, ZT7-12, ZT13-18, ZT19-24 (ZT1-6: t = 1.372, df = 92, p = 0.1735; ZT7-12: t = 4.613, df = 92, ***p<0.0001; ZT13-18: t = 0.2827, df = 92, p = 0.7780; ZT19-24: t = 9.590, df = 92, ***p<0.0001 by unpaired t-test). n = 47 in each group. (F) Sleep bout duration in *dbt*^S^ flies through a day (ZT1-6: t = 3.222, df = 91, **p = 0.0018; ZT7-12: t = 2.922, df = 91, **p = 0.0044; ZT13-18: t = 2.437, df = 91, *p = 0.0168; ZT19-24: t = 2.936, df = 91, **p = 0.0042 by unpaired t-test). n = 47 in each group. (G) Activity counts/awake time of *dbt*^S^ though a day (ZT1-6: t = 1.498, df = 92, p = 0.1375; ZT7-12: t = 1.441, df = 92, p = 0.1530; ZT13-18: t = 2.184, df = 92, *p = 0.0315; ZT19-24: t = 1.044, df = 90, p = 0.2993 by unpaired t-test). n = 47 in each group. (H) Total sleep amount during 24hrs (t = 5.379, df = 92, ***p<0.0001 by unpaired t-test). n = 47 each group. (I) Total sleep in *dbt*^L^ flies through a day. The four groups of bars are representing the total sleep of CS (black bar) and *dbt*^L^ mutants (purple bar) in ZT1-6, ZT7-12, ZT13-18, ZT19-24 (ZT1-6: t = 5.865, df = 92, ***p <0.0001, ZT7-12: t = 13.45, df = 92, ***p<0.0001, ZT13-18: t = 4.115, df = 92, ***p <0.0001, ZT19-24: t = 2.704, df = 92, **p = 0.0082 by unpaired t-test). n = 47 in each group. (J) Sleep bout duration in *dbt*^L^ flies through a day (ZT1-6: t = 2.814, df = 92, **p = 0.0060, ZT7-12: t = 8.175, df = 92, ***p<0.0001, ZT13-18: t = 2.103, df = 92, *p = 0.0382, ZT19-24: t = 2.059, df = 92, *p = 0.0423 by unpaired t-test). n = 47 in each group. (K) Activity counts/awake time of *dbt*^L^ through a day (ZT1-6: t = 6.600, df = 92, ***p <0.0001, ZT7-12: t = 2.581, df = 79, *p = 0.0117, ZT13-18: t = 6.049, df = 92, ***p <0.0001, ZT19-24: t = 3.128, df = 90, **p = 0.0024 by unpaired t-test). n = 47 in each group. (L) Total sleep amount in 24hrs (t = 1.762, df = 92, p = 0.0813 by unpaired t-test). n = 47 in each group. (M, N and O) Average actograms of CS, *dbt*^S^ and *dbt*^L^ flies in L12:D12 for 1 day and constant darkness for 5 days. Light represents day and grey represents night (n = 16 in each group). (P, Q and R) Average locomotor records of CS, *dbt*^S^ and *dbt*^L^ flies during L12:D12 (LD) for 1 day (CS, n = 47; *dbt*^S^, n = 46; *dbt*^L^, n = 48). ns. no significant difference, *p< 0.05, **p< 0.01 and ***p< 0.001. Black, orange and purple bars (or lines) respectively represent data of wildtype, *dbt*^S^ mutant and *dbt*^L^ mutant. The horizontal bar below each graph presents day (white) and night (black).

Although different locomotor activities were produced by these two mutants (see Fig 1M–1O), both of them show a sleep decrease during the nighttime. In addition, during the daytime, a decreased sleep amount was seen in *dbt*^S^ mutants while *dbt*^L^ males showed an increase (Fig 1A–1D). As the sleep phenotypes of both male and female flies are quite consistent with each other, the profiles shown here are for males only. We provide examples of female profiles in supplemental data (S1 Fig).

As these two mutants have different circadian phase, we can see a clear phase change in the sleep profile of these two mutants which is consistent with their circadian locomotor activity phase (Fig 1A and 1C; advanced in *dbt*^*S*^ and delayed in *dbt*^*L*^). Since the morning and evening peaks of activity in the *dbt*^S^ mutant are advanced while slightly delayed in the *dbt*^L^ mutant (Fig 1P–1R), the normal sleep totals for 12 hours daytime sleep and 12 hours nighttime sleep may not represent the phase of sleep properly in *dbt* mutants. Therefore, we divided one day into 4 periods of 6 hours and analyzed the sleep parameters in *dbt*^S^ and *dbt*^L^ mutants respectively. The results show that *dbt*^S^ has a significantly lower sleep amount and sleep bout duration in both late day (ZT7-12) and late night (ZT19-24) (Fig 1E and 1F). Importantly, sleep bout duration is reduced in early day (ZT1-6) and early night (ZT13-18), while overall levels of sleep are unchanged (ZT1-6 & ZT13-18 in Fig 1E and 1F), suggesting that the *dbt*^S^ mutation decreases sleep quality independently of total sleep amounts. Analysis of activity counts while the flies are awake reveals that the reductions of sleep observed during ZT7-12 and ZT19-24 are not due to hyperactive locomotor activity (Fig 1G). As for the *dbt*^L^ mutants, total sleep amount is decreased during ZT1-6, ZT13-18 and ZT19-24, and increased from ZT7-12 (Fig 1I). Importantly, reduced and elevated levels of sleep are associated with reduced sleep consolidation during ZT1-6, ZT13-18 and ZT19-24, and elevated sleep consolidation during ZT7-12 respectively (Fig 1J). Importantly, analysis of activity counts while awake indicates that reduction or increases in sleeping levels are not due to hyper- or hypoactivity respectively (Fig 1K).

There is a possibility that the sleep decrease of *dbt*^S^ mutants is caused by a circadian phase advance, since in *dbt*^S^ flies the sleep loss during the night happens in the late night from around ZT19-ZT24 (Fig 1A and 1E), and previous work showed that *dbt*^S^ flies have a short subjective day but a normal subjective night. This could mean that the night period for *dbt*^S^ flies is advanced by nearly 6 hours in constant darkness [9], and there may be a corresponding phase advance in LD, leading to the daytime sleep decrease after midday, the early sleep increase seen during late day in the profile, and the early sleep decrease seen after midnight.

As for *dbt*^L^ mutant, the phase of the sleep cycle is delayed, with persistent sleep happening in the late day and sleep loss happening in the early morning and early evening (Fig 1I). While these sleep assays are conducted in a 12hr:12hr LD cycle, nevertheless the *dbt*^S^ genotype has an advanced nighttime sleep phase which appears to start at ZT6-7, and *dbt*^L^ flies have a slightly delayed nighttime phase which starts after ZT12.

When we calculate the total sleep during a day of these mutants, we find that *dbt*^S^ flies have lower sleep amounts than wildtype flies while total sleep in *dbt*^L^ flies shows no difference with wildtype flies (Fig 1H and 1L). These results likely show that the sleep phenotype is caused by the locomotor activity phase change in *dbt* mutants. To better understand the effects of *dbt* on sleep, we decided to decrease the expression of *dbt* endogenously.

### Decreased expression of *dbt* throughout its multiple expression sites in the brain causes increased daytime sleep and decreased nighttime sleep

**Fig 2:**
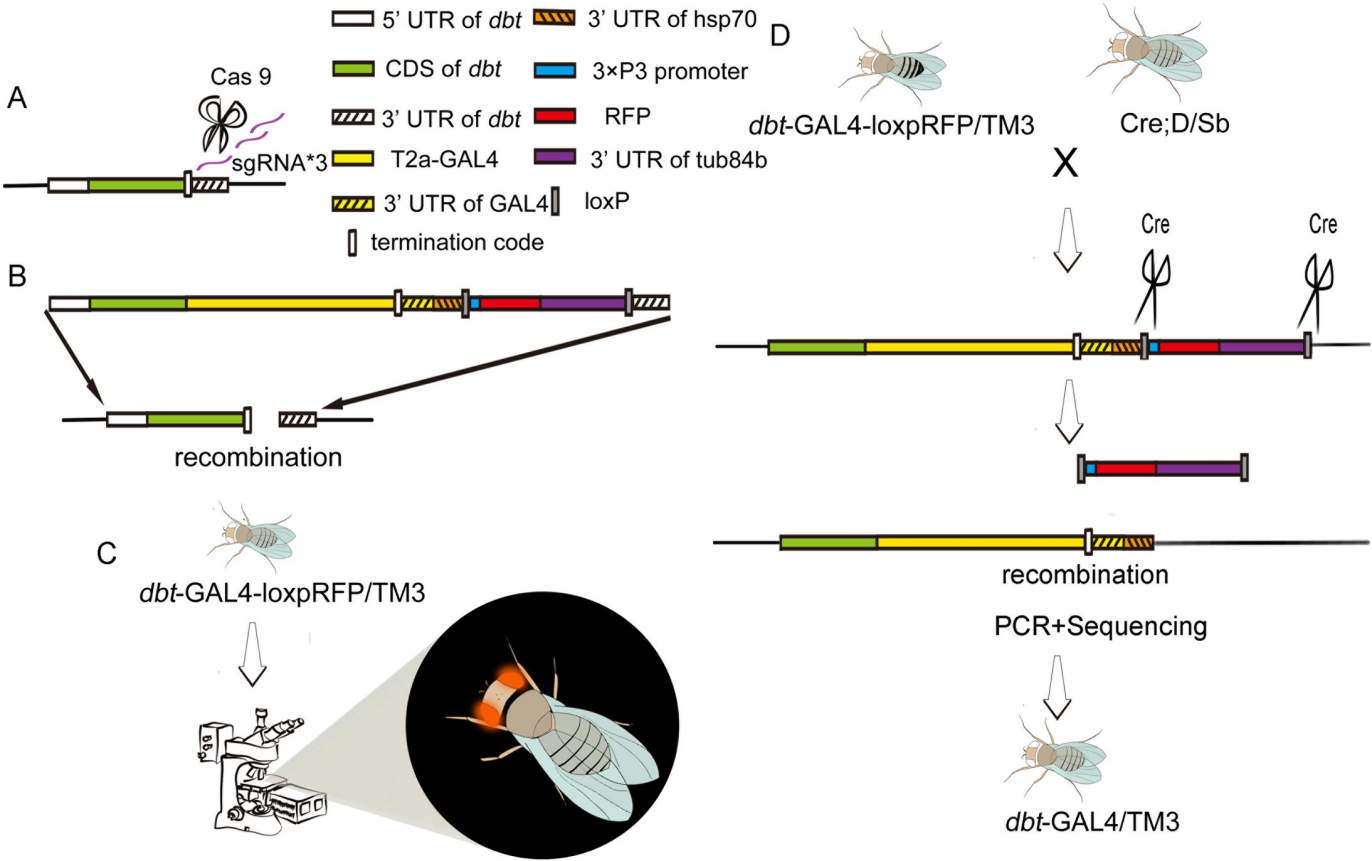
Construction of *dbt*-GAL4 line. (A) Cas-9 was used to identify and cut specific sites according to sequence of gRNAs. (B) The donor was constructed by connecting the 5’UTR of *dbt*+CDS of *dbt*, T2a-GAL4+3’UTR of GAL4+, 3’UTR of hsp70, loxP+3*P3 promoter +RFP+3’UTR of tub84b+loxP and 3’UTR of *dbt* into pBluescript-SK (+) vector. Then sequences in the plasmid which are same as the genomic gene can recombine with the specific sites which were cut by Cas-9. (C) The potential *dbt*-GAL4-RFP flies were screened by the RFP marker, which could be observed in the eyes of flies under a laser confocal microscope. (D) The Cre; D/Tm3Sb line was used to get rid of RFP by recombination. Then the *dbt*-GAL4 line was screened by PCR sequencing.

**Fig 3:**
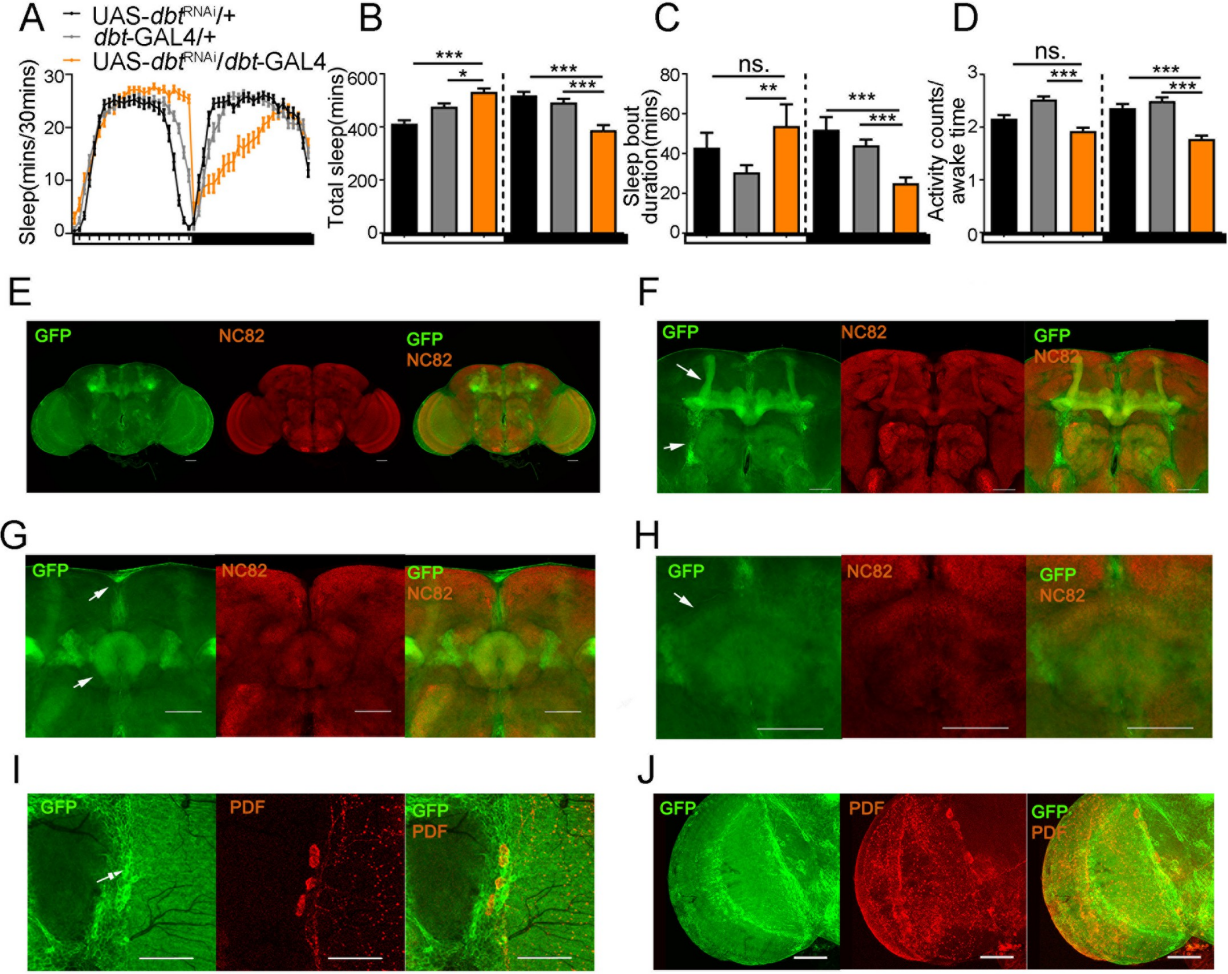
An increased daytime sleep and decreased nighttime sleep can be caused by decreasing the expression of *dbt* with *dbt*-GAL4, which is expressed broadly in the optic lobes, olfactory lobe and central brain, including the mushroom body, fan-shaped body, ellipsoid body, and pars intercerebralis. **Green: mCD8: GFP. Red: NC82 or PDF.** (A) Sleep profiles of flies with down-regulated *dbt* expression from *dbt-*GAL4 driver. n (UAS-*dbt*^RNAi^/+) = 42, n (*dbt*-GAL4/+) = 47, n (UAS-*dbt*^RNAi^/*dbt*-GAL4) = 24. (B) Average sleep amount during daytime and nighttime with down-regulated *dbt* expression from *dbt*-GAL4 driver. n (UAS-*dbt*^RNAi^/+) = 42, n (*dbt*-GAL4/+) = 47, n (UAS-*dbt*^RNAi^/*dbt*-GAL4) = 24. (C) Average sleep bout duration during daytime and nighttime with down-regulated *dbt* expression from *dbt*-GAL4 drivers. n (UAS-*dbt*^RNAi^/+) = 42, n (*dbt*-GAL4/+) = 47, n (UAS-*dbt*^RNAi^/*dbt*-GAL4) = 24. (D) Activity counts/awake time with down-regulated *dbt* expression from *dbt*-GAL4 drivers. n (UAS-*dbt*^RNAi^/+) = 42, n (*dbt*-GAL4/+) = 47, n (UAS-*dbt*^RNAi^/*dbt*-GAL4) = 24. (E) The expression pattern of *dbt*-GAL4 detected with UAS-mCD8: GFP and co-stained with NC82 in the whole brain. Scale bar = 50um. (F-H) Expression of UAS-mCD8: GFP driven by *dbt*-GAL4 and stained with anti-GFP (green) and anti-NC82 (red) to localize the expression of *dbt*. The localization shows that DBT protein is expressed broadly in the mushroom body (upper arrow in F), antennal lobe (lower arrows in F), pars intercerebralis (upper arrow in G), ellipsoid body (lower arrow in G), fan-shaped body (arrow in H). Scale bar = 50um. (I-J) Expression of UAS-mCD8: GFP driven by *dbt*-GAL4 and stained with anti-GFP (green) and anti-PDF (red) to localize the expression of *dbt*. The localization shows that DBT protein is also expressed in the lateral neurons (I: l-LNv marked with an arrow; s-LNv are PDF-positive neurons below these) and optic lobes (J). Scale bar = 50um. ns. no significant difference, *p< 0.05, **p< 0.01 and ***p< 0.001. Black and grey bars (or lines) respectively represent UAS-/+, *-*GAL4/+ control flies, while orange bars (or lines) represent the treatment flies. The horizontal bar below each graph presents day (white) and night (black).

To decrease the expression of *dbt* endogenously, we constructed a *dbt*-GAL4 line by inserting the T2AGAL4 reading frame fused in frame immediately downstream of the last amino acid in *dbt* (Fig 2). Then we used the UAS/GAL4 system to express UAS-*dbt*^RNAi^ with our newly created *dbt-*GAL4 driver. Not surprisingly, we found that only a few flies with the UAS-*dbt*^RNAi^/*dbt*-GAL4 genotype emerged from around 30–50 pupae, illustrating the key role played by *dbt* during development. From the emerging progeny, after 3–5 days of sleep evaluation, flies with general down-regulation of *dbt* exhibited a sleep increase during the daytime and a sleep decrease during the nighttime (Fig 3A and 3B). Interestingly, daytime sleep bout duration is no different while nighttime sleep bout duration is significantly decreased when compared with the 2 controls (Fig 3C). To exclude the possibility that the sleep increase observed during the daytime in *dbt*-GAL4/UAS-*dbt*^RNAi^ flies is caused by lower activity, we calculated the activity counts/awake time. Fig 3D shows that the locomotor activity of *dbt*-GAL4/UAS-*dbt*^RNAi^ does not differ significantly from controls during daytime. Importantly, the sleep decrease observed during the night in *dbt*-GAL4/UAS-*dbt*^RNAi^ flies is not caused by hyperactivity (Fig 3D). These results suggest that sleep changes seen in *dbt*-GAL4/UAS-*dbt*^RNAi^ flies are not caused by abnormal locomotor activity, and the overall phase difference in the sleep profile relative to controls suggests a delayed phase for the sleep profile. The delayed phase is most likely due to the long-period circadian phenotype for this genotype (Table 1).

**Table 1:** Activity rhythm of flies with down regulated *dbt*.

Genotype	total flies	Rhythmic flies (%)	Period (h)
UAS-*dbt*^RNAi^/+♂	34	85.20	24.53±0.04
*dbt*-GAL4/+♂	35	88.57	24.59±0.07
UAS-*dbt*^RNAi^/ *dbt*-GAL4	27	57.14	25.44±0.15
UAS-*dicer*/+; UAS-*dbt*^RNAi^/+♂	45	93.93	23.99±0.04
*tim-*GAL4/+♂	44	91.00	24.10±0.06
UAS-*dicer*/+; *tim-*GAL4/+;UAS-*dbt*^RNAi^/+♂	43	22.57	26.22±1.43–
*per-*GAL4/+♂	48	89.63	24.26±0.09
UAS-*dicer*/*per-*GAL4; UAS-*dbt*^RNAi^/+♂	43	89.63	23.98±0.05
*201Y/+*♂	47	93.63	24.19±0.06
201Y/UAS-*dicer*; UAS-*dbt*^RNAi^/+♂	46	80.00	24.08±0.07
C309/+♂	47	91.10	23.83±0.29
C309/UAS-*dicer*; UAS-*dbt*^RNAi^*/+*♂	45	93.50	24.15±0.07
C205/*+*♂	47	95.70	23.87±0.08
UAS-*dicer*/+; UAS-*dbt*^RNAi^/C205♂	44	93.20	23.67±0.30
C819/*+*♂	47	89.20	24.04±0.03
UAS-*dicer*/+; UAS-*dbt*^RNAi^/C819♂	48	95.83	24.03±0.04

*All the flies were detected in the constant darkness for 5–7 days after entrainment in an LD cycle for 3 days. The rhythmic rate and period are analyzed by the faasX software.

While the DBT protein in the *dbt*^S^, *dbt*^L^ and *dbt*^RNAi^/*dbt*-GAL4 genotypes is expressed in all cells in which *dbt* is normally expressed, the total extent of *dbt* expression in the brain of adult flies is still unclear. Previous research showed that *dbt* transcripts were expressed in both the optic lobes and some central neurons [7,19]. In order to localize the expression of *dbt*, a UAS-mCD8: GFP reporter was expressed with the *dbt*-GAL4 driver. Immunostaining with anti GFP, anti NC82 and anti PDF antibodies show that *dbt*-GAL4 is expressed broadly in the optic lobes (Fig 3E and 3J), antennal lobe (lower arrow in Fig 3F) and the central brain, including the mushroom bodies (upper arrow in Fig 3F), ellipsoid body (lower arrow in Fig 3G), fan-shaped body (arrow in Fig 3H), and pars intercerebralis (upper arrow in Fig 3G). It is also detected in the large and small LNvs, whose cell bodies express PDF (Fig 3I).

### *Dbt* affects daytime sleep in a manner that is dependent on developmental processes

**Fig 4:**
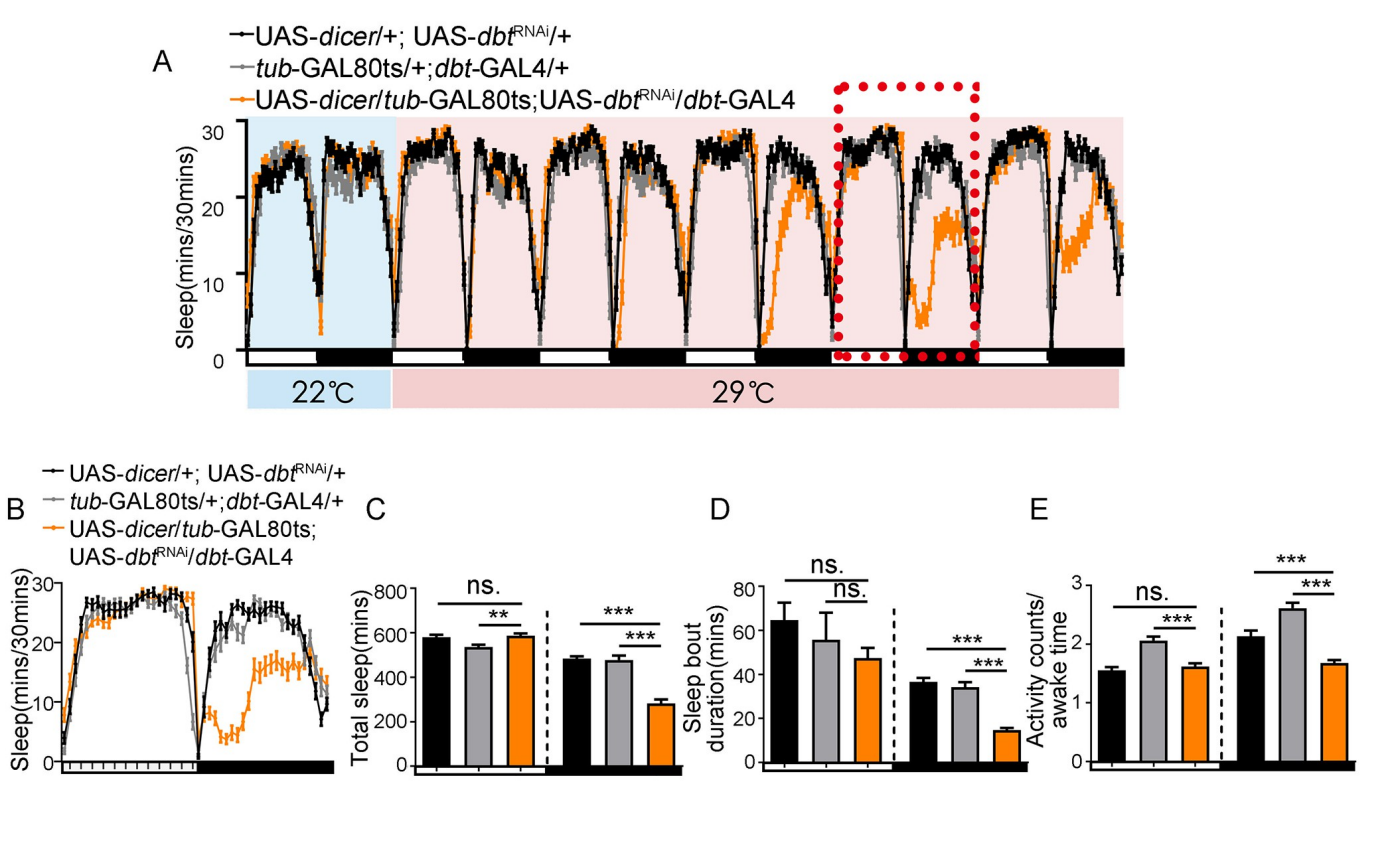
Sleep phenotypes of flies with *dbt* down regulation only during adulthood. (A) Sleep profile of flies with adult specific reduction of *dbt* level. The flies were raised at 18°C and sleep was detected at 22°C for 2 days (blue background) and 29°C for 5 days (pink background). The sleep parameters of the day which is marked with red-dotted frame is shown in B-E. n (UAS-d*icer*/+; UAS-*dbt*^RNAi^) = 44, n (*tub*-GAL80ts/+; *dbt*-GAL4/+) = 45, n (UAS-*dicer*/*tub*-GAL80ts; UAS-*dbt*^RNAi^/*dbt*-GAL4) = 47. (B) Sleep profile of flies with adult specific reduction of *dbt* level; this is taken from the day marked with the red-dotted frame above. n (UAS-d*icer*/+; UAS-*dbt*^RNAi^) = 44, n (*tub*-GAL80ts/+; *dbt*-GAL4/+) = 45, n (UAS-*dicer*/*tub*-GAL80ts; UAS-*dbt*^RNAi^/*dbt*-GAL4) = 47. (C) Total sleep of flies with lower *dbt* in adult stage. The total sleep was significantly decreased during the nighttime with no significant difference during the daytime. n (UAS-d*icer*/+; UAS-*dbt*^RNAi^) = 44, n (*tub*-GAL80ts/+; *dbt*-GAL4/+) = 45, n (UAS-*dicer*/*tub*-GAL80ts; UAS-*dbt*^RNAi^/*dbt*-GAL4) = 47. (D) Sleep bout duration of flies with lower *dbt* in adult stage. n (UAS-d*icer*/+; UAS-*dbt*^RNAi^) = 44, n (*tub*-GAL80ts/+; *dbt*-GAL4/+) = 45, n (UAS-*dicer*/*tub*-GAL80ts; UAS-*dbt*^RNAi^/*dbt*-GAL4) = 47. (E) Activity counts/awake time of flies with lower *dbt* in adult stage. n (UAS-d*icer*/+; UAS-*dbt*^RNAi^) = 44, n (*tub*-GAL80ts/+; *dbt*-GAL4/+) = 45, n (UAS-*dicer*/*tub*-GAL80ts; UAS-*dbt*^RNAi^/*dbt*-GAL4) = 47. ns. no significant difference, *p< 0.05, **p< 0.01 and ***p< 0.001. Black and grey bars (or lines) respectively represent UAS-/+, *-*GAL4/+ control flies, while orange bars (or lines) represent the treatment flies. The horizontal bar below each graph presents day (white) and night (black).

*Dbt* is also known as *disc overgrown* (*dco*), and besides its function in circadian rhythms, it also involved in some developmental processes, such as the Wnt-TCF signaling pathway and the Hippo signaling pathway [13,20,21], so there is a possibility that the effect of *dbt* on sleep may depend on its function in developmental processes. To determine if *dbt* affects sleep through its effects on developmental processes, we constructed a stable line with both *tub*-GAL80ts and *dbt*-GAL4, and down-regulated *dbt* with this line only in the adult or larval stage. In order to inhibit the effect of GAL4 during development, we raised the flies at 18°C, and analyzed adult sleep at 22°C for one day and then elevated the temperature to 29°C to knock down *dbt* (Fig 4A). Results showed a significant decrease in nighttime sleep after 3 days at 29 degrees accompanied by a lower sleep bout duration (Fig 4B–4D), but no change during daytime after several days of *dbt* knock-down (Fig 4A–4C). The reduction of sleep observed during nighttime is not due to hyperactive locomotor activity (Fig 4E). Consistent results were also obtained in *tub*-GAL80ts/+; UAS-*dbt*^K/R^/*dbt*-GAL4 flies overexpressing a dominant negative DBT^K/R^ protein [11] and occurred immediately after the elevation to 29°C (S2 Fig), most likely because overexpression of the dominant negative DBT^K/R^ occurred immediately and did not take as long as the RNAi-mediated knock-down of DBT. On the other hand, when we raised *tub*-GAL80ts/UAS-*dicer*; UAS-*dbt*^RNAi^/*dbt-*GAL4 flies at 29°C to down-regulate *dbt* expression during development, no adult flies emerged (S1 Table). These data indicate that the effect of *dbt* on daytime sleep is probably due to developmental processes. Importantly, the effect on nighttime sleep is caused by loss of *dbt* in the adult.

### Loss of expression of *dbt* in clock neurons causes a sleep decrease during the nighttime, while loss of expression in other neurons leads to elevated daytime sleep

DBT is a kinase that plays many different roles in various biological processes [7–16]. To limit its function in specific tissues, and to examine the circadian effect of *dbt* on the sleep phenotype, we used the UAS/GAL4 system to express UAS-*dbt*^RNAi^ with two clock neuron drivers—*tim-*GAL4 and *per-*GAL4. A UAS-*dicer* was separately included in the *tim-*GAL4 or *per-*GAL4 and UAS-*dbt*^RNAi^ to enhance the effect of UAS-*dbt*^RNAi^.

**Fig 5:**
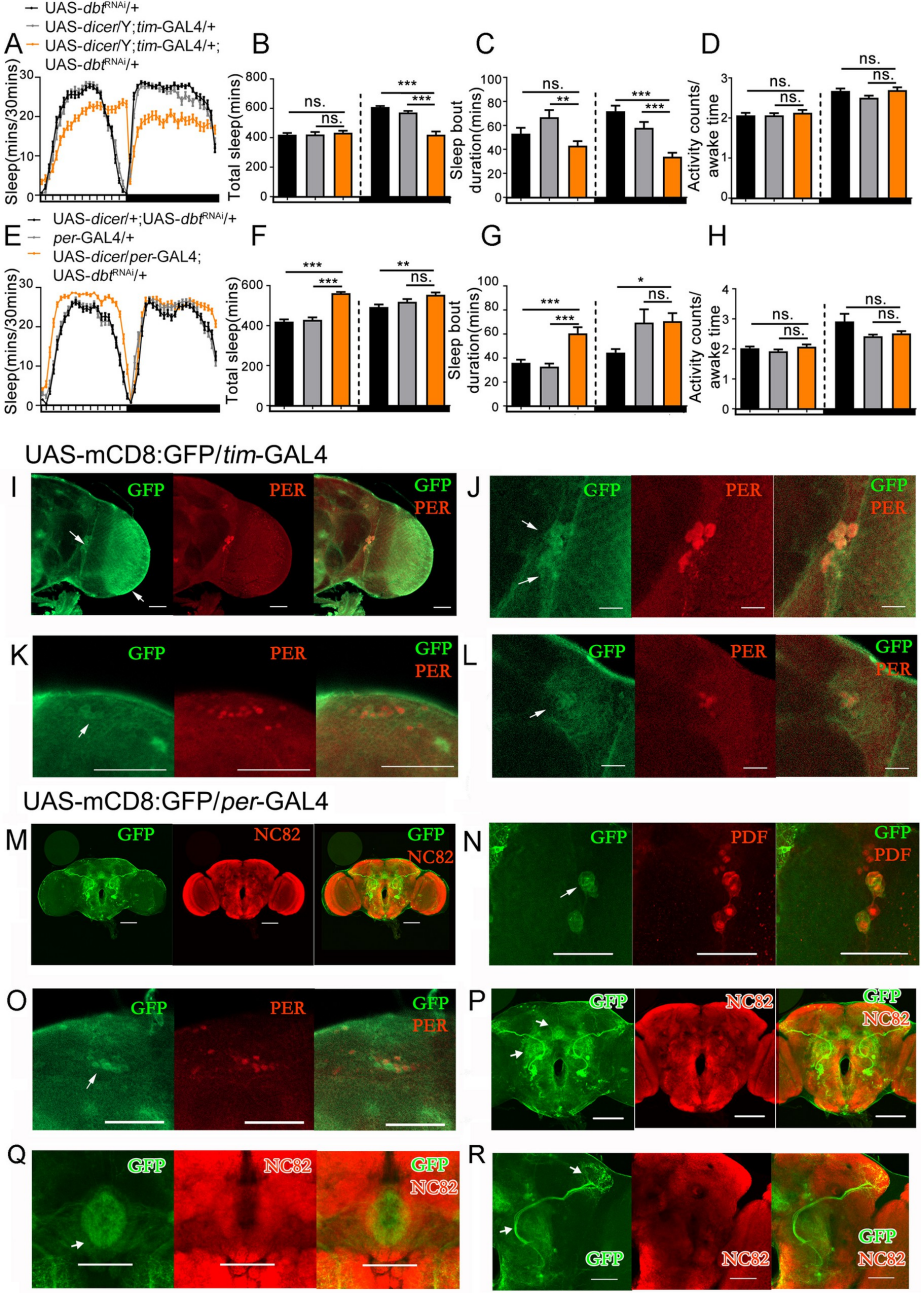
Loss of expression of *dbt* in *tim-*GAL4-expressing neurons can lead to a sleep decrease during the nighttime, while loss of expression of *dbt* with *per-*GAL4 can lead to a sleep increase during the daytime. (A and E) Sleep profiles of male flies with down-regulated *dbt* expression from *tim-*GAL4 and *per-*GAL4. n (UAS-*dbt*^RNAi^/+) = 46, n (UAS-*dicer*/Y; *tim*-GAL4/+) = 48, n (UAS-*dicer*/Y; *tim*-GAL4/+; UAS-*dbt*^RNAi^/+) = 48 in A; n (UAS-*dicer*/+; UAS-*dbt*^RNAi^/+) = 48, n (*per*-GAL4/+) = 45, n (UAS-*dicer*/*per*-GAL4; UAS-*dbt*^RNAi^/+) = 48 in E. (B) Average sleep amount during daytime and nighttime with down-regulated *dbt* expression from *tim-*GAL4 driver. n (UAS-*dbt*^RNAi^/+) = 46, n (UAS-*dicer*/Y; *tim*-GAL4/+) = 48, n (UAS-*dicer*/Y; *tim*-GAL4/+; UAS-*dbt*^RNAi^/+) = 48. (C) Average sleep bout duration during daytime and nighttime with down-regulated *dbt* expression from *tim-*GAL4 driver. n (UAS-*dbt*^RNAi^/+) = 46, n (UAS-*dicer*/Y; *tim*-GAL4/+) = 48, n (UAS-*dicer*/Y; *tim*-GAL4/+; UAS-*dbt*^RNAi^/+) = 48. (D) Activity counts/awake time with down-regulated *dbt* expression from *tim-*GAL4 driver. n (UAS-*dbt*^RNAi^/+) = 46, n (UAS-*dicer*/Y; *tim*-GAL4/+) = 48, n (UAS-*dicer*/Y; *tim*-GAL4/+; UAS-*dbt*^RNAi^/+) = 48. (F) Average sleep amount during daytime and nighttime with down-regulated *dbt* expression from *per-*GAL4 driver. n (UAS-*dicer*/+; UAS-*dbt*^RNAi^/+) = 48, n (*per*-GAL4/+) = 45, n (UAS-*dicer*/*per*-GAL4; UAS-*dbt*^RNAi^/+) = 48. (G) Average sleep bout duration during daytime and nighttime with down-regulated *dbt* expression from *per-*GAL4 drivers. n (UAS-*dicer*/+; UAS-*dbt*^RNAi^/+) = 48, n (*per*-GAL4/+) = 45, n (UAS-*dicer*/*per*-GAL4; UAS-*dbt*^RNAi^/+) = 48. (H) Activity counts/awake time with down-regulated *dbt* expression from *per-*GAL4 driver. n (UAS-*dicer*/+; UAS-*dbt*^RNAi^/+) = 48, n (*per*-GAL4/+) = 45, n (UAS-*dicer*/*per*-GAL4; UAS-*dbt*^RNAi^/+) = 48. (I-L) The expression patterns of *tim-*GAL4 detected with UAS-mCD8-GFP and stained with anti-GFP (green) and anti-PER (red). The *tim-*GAL4 is expressed in the clock neurons, including the L-LNvs (upper arrow in I and J), optic lobe (lower arrow in I), s-LNv (lower arrow in J), DNs (K) and LNds (L). Scale bar = 50um. (M-R) The expression patterns of *per-*GAL4 detected with UAS-mCD8-GFP and stained with anti-GFP (green in M-R), anti-PDF (red in N), anti-PER (red in O) and anti NC82 (red in M & P-R). The *per-*GAL4 is not only expressed in parts of LNvs (arrow in N), DNs (arrow in O) but also in other areas including the ellipsoid body (upper arrow in P and arrow in Q), antennal lobe (AL) (lower arrow in P), medio-lateral antennal lobe tract (lower arrow in R), and lateral horn (upper arrow in R). Scale bar = 50um. ns. no significant difference, *p< 0.05, **p< 0.01 and ***p< 0.001. Black and grey bars (or lines) respectively represent UAS-/+, *-*GAL4/+ control flies, while orange bars (or lines) represent the treatment flies. The horizontal bar below each graph presents day (white) and night (black).

Down-regulation of *dbt* expression with a *tim-*GAL4 driver causes a sleep decrease during the nighttime in LD (Fig 5A and 5B) but also causes a reduced rhythmicity (22.6%) and a longer period (26.2hrs) in constant darkness (DD) (Table 1). However, with the *per-*GAL4 driver, sleep was increased during the daytime with no significant difference during the night time (Fig 5E and 5F), and these changes were reflected with a change of sleep bout duration (a decrease with *tim*-GAL4 at night and an increase with *per-*GAL4 during the day) (Fig 5C and 5G). Note that even though down-regulation of *dbt* expression with *per-*GAL4 or *tim*-GAL4 alters sleep, the locomotor activity of these flies during awake time has no statistically significant difference from controls, meaning that the altered sleep of these flies is not due to the difference between their activities (Fig 5D and 5H).

It is possible that these different results are caused by the different expression patterns of the two GAL4 drivers. To verify expression patterns of the two drivers in the fly brain, we crossed each of them with a UAS-mCD8: GFP reporter, and their expression patterns in the brain were detected after dissecting and immunostaining with anti GFP. After microscopy, we find that *tim-*GAL4 is clearly expressed mostly in the circadian neurons (Fig 5I–5L) including the l-LNv and s-LNv (Fig 5J), DNs (Fig 5K), optic lobe (lower arrow in Fig 5I) and LNd (arrow in Fig 5L), while *per-*GAL4 is expressed widely in the brain (Fig 5M–5R), including in two lateral neurons (LNvs) (arrow in Fig 5N) and dorsal neurons (DNs) (arrow in Fig 5O), ellipsoid body (EB) (upper arrow in Fig 5P and 5Q), antennal lobe (AL) (lower arrow in Fig 5P), medio-lateral antennal lobe tract (lower arrow in Fig 5R) and lateral horn (upper arrow in Fig 5R).

In order to exclude the possibility that *dbt* may affects sleep through glial cells, in which *tim*-GAL4 is expressed [22], we used the glial-specific *repo*-GAL4 driver to reduce the expression of *dbt* in glial cells and tested the sleep phenotype. Results showed that the sleep amount of UAS-*dicer*; UAS-*dbt*^RNAi^/*repo*-GAL4 did not have a significant difference with the control flies (S3I and S3J Fig). Thus, according to the previous reports and result in this paper, we find that the sleep difference of UAS-*dicer*; UAS-*dbt*^RNAi^/*tim*-GAL4 was because of the lower expression of *dbt* in clock neurons rather than glial cells or any other neurons. Given the different effects that down-regulation of *dbt* with *per-*GAL4 and *tim-*GAL4 have on sleep, *dbt* affects sleep through both clock neurons and non-clock neurons.

### Overexpression of *dbt*^K/R^ with the *tim-*GAL4 driver leads to a sleep decrease during the nighttime

**Fig 6:**
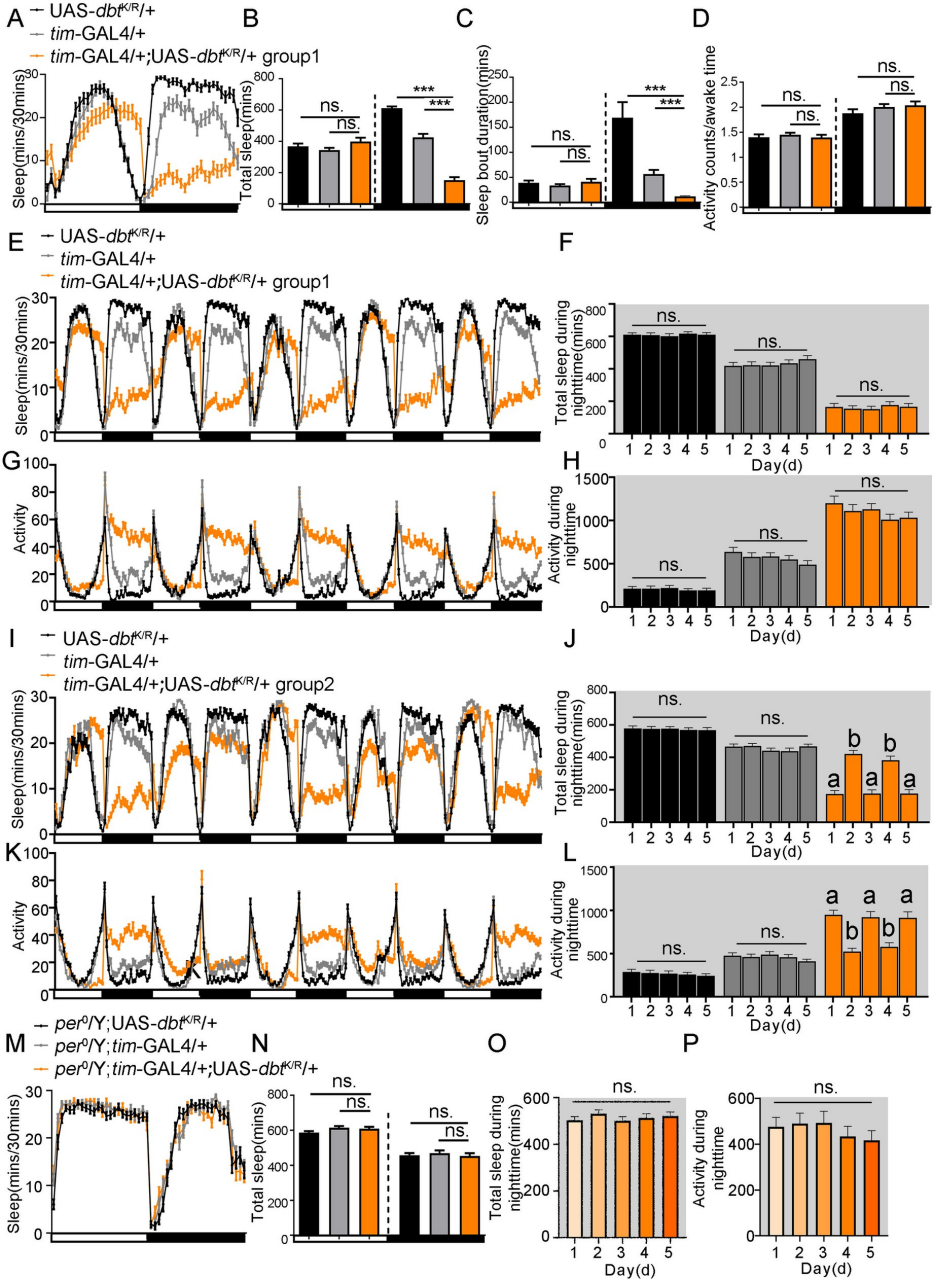
Overexpression of *dbt*^K/R^ with the *tim-*GAL4 driver leads to a sleep decrease during the nighttime. (A**)** Sleep profile of flies with high expression of *dbt*^K/R^ in clock neurons. n (UAS-*dbt*^K/R^/+) = 42, n (*tim*-GAL4/+) = 48, n (*tim-*GAL4/+; UAS-*dbt*^K/R^/+ group1) = 48. (B) Average sleep amount during daytime and nighttime with high expression of *dbt*^K/R^ from *tim-*GAL4 driver. n (UAS-*dbt*^K/R^/+) = 42, n (*tim*-GAL4/+) = 52, n (*tim-*GAL4/+; UAS-*dbt*^K/R^/+ group1) = 51. (C) Average sleep bout duration during daytime and nighttime with high expression of *dbt*^K/R^ from *tim-*GAL4 driver. n (UAS-*dbt*^K/R^/+) = 42, n (*tim*-GAL4/+) = 52, n (*tim-*GAL4/+; UAS-*dbt*^K/R^/+ group1) = 51. (D) Activity counts/awake time with high expression of *dbt*^K/R^ from *tim-*GAL4 driver. n (UAS-*dbt*^K/R^/+) = 42, n (*tim*-GAL4/+) = 52, n (*tim-*GAL4/+; UAS-*dbt*^K/R^/+ group1) = 51. (E) 5 days sleep profiles of flies with high expression of *dbt*^K/R^ in clock neurons (group1). n (UAS-*dbt*^K/R^/+) = 42, n (*tim*-GAL4/+) = 52, n (*tim-*GAL4/+; UAS-*dbt*^K/R^/+ group1) = 51. (F) Average sleep amount during the nighttime of flies with overexpression of *dbt*^K/R^ in clock neurons in 5 days (group1). n (UAS-*dbt*^K/R^/+) = 42, n (*tim*-GAL4/+) = 52, n (*tim-*GAL4/+; UAS-*dbt*^K/R^/+ group1) = 51. (G) Activity profiles of flies with high expression of *dbt*^K/R^ in clock neurons in 5 days (group1). n (UAS-*dbt*^K/R^/+) = 42, n (*tim*-GAL4/+) = 52, n (*tim-*GAL4/+; UAS-*dbt*^K/R^/+ group1) = 51. (H) Activity counts during the nighttime of flies with overexpression of *dbt*^K/R^ in clock neurons in 5 days (group1). n (UAS-*dbt*^K/R^/+) = 42, n (*tim*-GAL4/+) = 52, n (*tim-*GAL4/+; UAS-*dbt*^K/R^/+ group1) = 51. (I) 5 days sleep profiles of flies with high expression of *dbt*^K/R^ in clock neurons (group2). n (UAS-*dbt*^K/R^/+) = 47, n (*tim*-GAL4/+) = 45, n (*tim-*GAL4/+; UAS-*dbt*^K/R^/+ group2) = 46. This is an average of individual male UAS-*dbt*^*K/R*^/+;*timGAL4*/+ flies that exhibited alternating high and low sleep amounts on successive nights. (J) Average sleep amount during the nighttime in flies with overexpressing *dbt*^K/R^ in clock neurons for 5 days (group2). n (UAS-*dbt*^K/R^/+) = 47, n (*tim*-GAL4/+) = 45, n (*tim-*GAL4/+; UAS-*dbt*^K/R^/+ group2) = 46. (K) Activity profiles of flies with high expression of *dbt*^K/R^ in clock neurons in 5 days (group2). n (UAS-*dbt*^K/R^/+) = 47, n (*tim*-GAL4/+) = 45, n (*tim-*GAL4/+; UAS-*dbt*^K/R^/+ group2) = 46. (L) Activity counts during the nighttime of flies with overexpression of *dbt*^K/R^ in clock neurons in 5 days (group2). n (UAS-*dbt*^K/R^/+) = 47, n (*tim*-GAL4/+) = 45, n (*tim-*GAL4/+; UAS-*dbt*^K/R^/+ group2) = 46. (M) Sleep profile of flies with high expression of *dbt*^K/R^ in clock neurons without expression of PER protein. n (*per*^0^/Y; UAS-*dbt*^K/R^/+) = 46, n (*per*^0^/Y; *tim-*GAL4/+) = 47, n (*per*^0^/Y; *tim-*GAL4/+; UAS-*dbt*^K/R^/+) = 45. (N) Average sleep amount during the daytime and nighttime in UAS-*dbt*^K/R^/*tim-*GAL4 flies without PER expression. n (*per*^0^/Y; UAS-*dbt*^K/R^/+) = 46, n (*per*^0^/Y; *tim-*GAL4/+) = 47, n (*per*^0^/Y; *tim-*GAL4/+; UAS-*dbt*^K/R^/+) = 45. (O) Average sleep amount of UAS-*dbt*^K/R^/*tim-*GAL4 flies during the nighttime without expression of PER for 5 days. n = 47. (P) Activity counts of UAS-*dbt*^K/R^/*tim-*GAL4 flies during the nighttime without expression of PER for 5 days. n = 47. ns. no significant difference, *p< 0.05, **p< 0.01 and ***p< 0.001 in B-D and N-P, the a, b represents *p< 0.05 between 2 of the groups in J and L. Black and grey bars (or lines) respectively represent UAS-/+, *-*GAL4/+ control flies, while orange or red bars (or lines) represent the treatment flies. The horizontal bar below some of graphs presents day (white) and night (black).

*Dbt*K38R (*dbt*^K/R^) is another site-specific mutation of *dbt*; the mutant protein can bind PER in vivo but lacks the protein kinase activity of DBT^WT^ and thereby inhibits the activity of DBT^WT^ by competitive binding to PER as a dominant negative. When *dbt*^K/R^ is expressed in clock neurons of the fly, a very long period or arrhythmicity is produced [11]. In this study, we find that down-regulation of *dbt* with the *tim-*GAL4 driver and RNAi can cause a sleep decrease during the nighttime. In order to verify this conclusion, we used a *tim-*GAL4 driver to drive the expression of UAS-*dbt*^K/R^ to determine if the same sleep phenotype is produced. As we expected, one group (group 1) of *tim*-GAL4/+; UAS-*dbt*^*K/R*^/+ flies show a sleep decrease during the nighttime, while its sleep amount during the daytime is not changed (Fig 6A and 6B), and the sleep bout duration of these flies is reduced during the nighttime (Fig 6C), a phenotype consistent with UAS-*dicer*/*tim*-GAL4; UAS-*dbt*^*RNAi*^/+ flies. Additionally, the activity during awake time of *tim*-GAL4/+; UAS-*dbt*^*K/R*^/+ flies is not changed, which means the sleep reduction of these flies during the nighttime is not due to hyperactivity (Fig 6D).

During 5 days of sleep monitoring, we also found another group of experimental flies (group 2: *tim-*GAL4/+; UAS-*dbt*^K/R^/+) showing a sleep change every single night (a cycle of high sleep one night and low sleep the next) (Fig 6I and 6J), a pattern which is slightly different from the *tim-*GAL4/+; UAS-*dbt*^K/R^/+ group 1 flies with a stable sleep decrease during the nighttime (Fig 6E and 6F). The *tim-*GAL4/+; UAS-*dbt*^K/R^/+ were from the same genotype and experiments, but they produced two different sleep phenotypes categorized into two groups, in which some independent repeats only exhibited one type of sleep phenotype (group 1), other independent repeats only exhibited another sleep phenotype (group 2), and some independent repeats simultaneously exhibited two kinds of phenotypes (i.e., group 1 and group 2). From the sleep profiles of single flies of *tim-*GAL4/+; UAS-*dbt*^K/R^/+, we found that the rate of the *tim-*GAL4/+; UAS-*dbt*^K/R^/+ (group 2) sleep change could range from around 20% to 50% from one night to the next.

As a previous study showed that overexpression of *dbt*^K/R^ produced either longer period flies or arrhythmic flies, we wondered if its effects on sleep are based on circadian differences. Therefore, we analyzed the locomotor activity of the 2 groups of *tim-*GAL4/+; UAS-*dbt*^K/R^/+ flies in LD condition, and their circadian rhythmicity and periods in DD condition. The results show that the activity of group 1 *tim-*GAL4/+; UAS-*dbt*^K/R^/+ flies has a stable higher activity during the nighttime (Fig 6G and 6H), while the activity of group 2 still changes from one night to the next, with a night of high activity followed by a night of low activity (Fig 6K and 6L). Therefore, the long-period oscillations of sleep are produced by long-period changes in activity levels. As for the detection of circadian rhythm and period of these flies, the results show that both groups of *tim-*GAL4/+; UAS-*dbt*^K/R^/+ have lower circadian rhythmicity and longer periods (Table 2), but group 2 *tim-*GAL4/+; UAS-*dbt*^K/R^/+ has an even longer circadian period and is more arrhythmic than group 1, potentially leading to a lack of entrainment of sleep to a 24 hr LD. Finally, the expression of a UAS-*dbt*^*WT*^ with either the *tim*-GAL4 or *per*-GAL4 driver has no effect on the sleep profile (S5 Fig)–a result consistent with its weak effects on circadian rhythms [11].

**Table 2:** Activity Rhythm of flies with over-expressed *dbt*^K/R^ in clock neurons which leads to a long-period oscillation or no oscillation of nighttime sleep amounts.

Genotype	total flies	Rhythmic flies (%)	Period (h)
*tim*-GAL4/+ ♂	70	89.52±0.09	24.06±0.02 a
UAS-*dbt*^K/R^/*tim*-GAL4 group1 with lower night sleep ♂	57	67.55±0.07	27.80±0.66 b
UAS-*dbt*^K/R^/*tim*-GAL4 group2 with long period oscillation of nighttime sleep ♂	50	39.50±0.05	30.25±0.91 c

*All the flies were detected in the constant darkness for 5–7 days after entrainment in an LD cycle for 3 days. The rhythmic rate and period are analyzed by the faasX software. The statistical analysis of the period was performed by GraphPad Prism 8. Anova F [2, 115] = 45.36, P<0.0001; the a, b, c periods differ significantly from each other.

Then we assessed if this phenotype requires PER to be manifested in *tim-*GAL4/+; UAS-*dbt*^K/R^/+. We expressed *dbt*^K/R^ with *tim-*GAL4 in *per*^0^ background and found that the sleep decrease during the nighttime of all days disappeared, while the sleep amounts were no different from the *per*^0^/Y; UAS-*dbt*^*K/R*^/+ and *per*^0^; *tim*GAL4/+ controls (Fig 6M and 6N). In addition, the variations of phenotype group 2 on nighttime sleep and activity in the *per*^0^/Y; UAS-*dbt*^K/R^/*tim*-GAL4 flies also disappeared (Fig 6O and 6P). These data indicate that the sleep phenotypes of *dbt*^K/R^ expression, with a decrease during the nighttime, is dependent on PER protein.

### *Dbt* affects daytime sleep through the central complex and mushroom body neurons in a manner that is also based on Period protein

**Fig 7:**
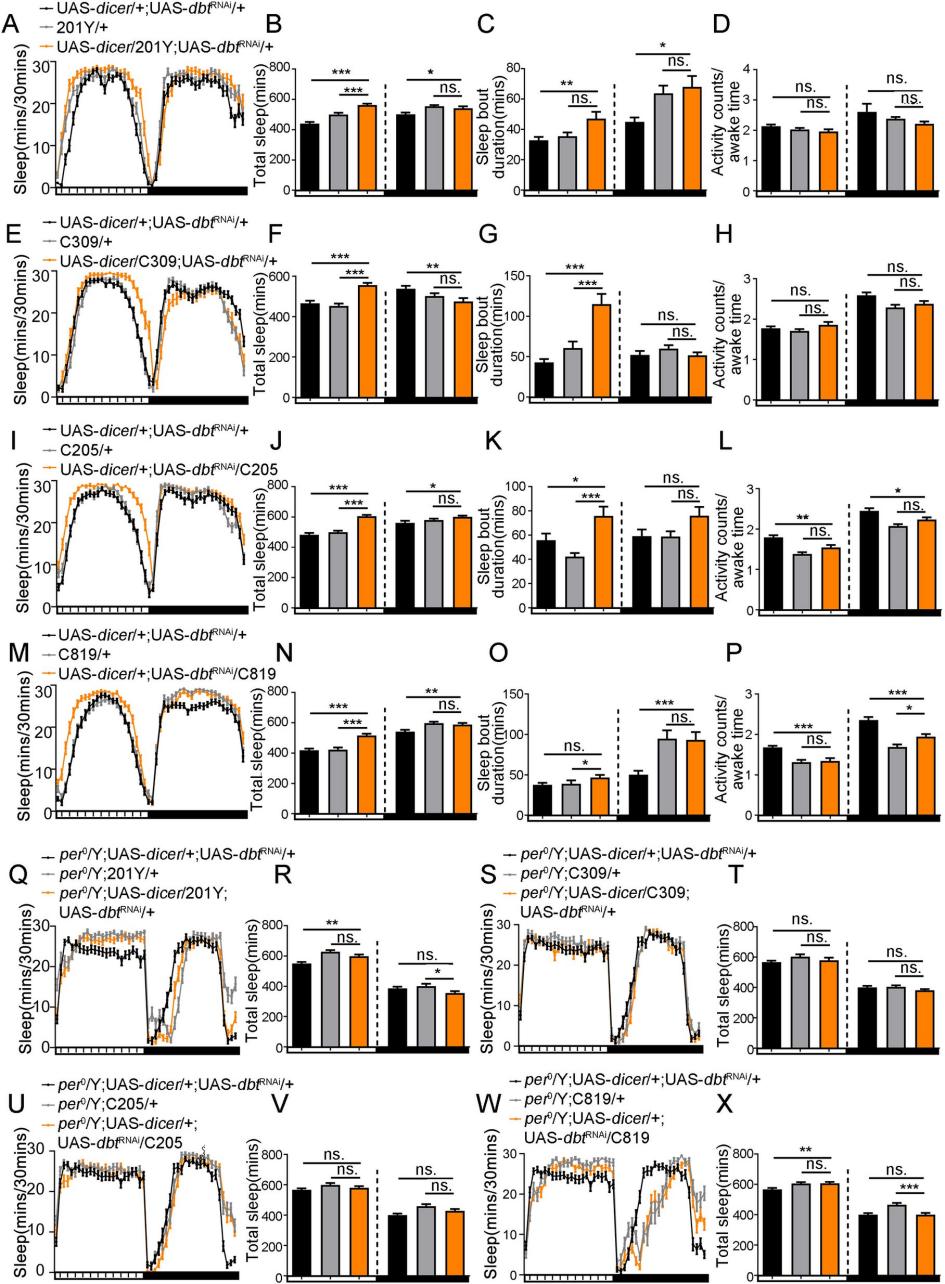
Knock down of *dbt* expression in mushroom body and central complex neurons caused a daytime sleep increase which is dependent on PER protein. (A) Loss of expression of *dbt* in α, β and γ lobes of mushroom body (201Y) of flies causes a sleep increase during the daytime. n (UAS-*dicer*/+; UAS-*dbt*^RNAi^/+) = 46, n (201Y/+) = 45, n (UAS-*dicer*/201Y; UAS-*dbt*^RNAi^/+) = 38. (B) Average sleep amount during the daytime and nighttime with down-regulation of *dbt* expression with mushroom body driver 201Y. n (UAS-*dicer*/+; UAS-*dbt*^RNAi^/+) = 46, n (201Y/+) = 45, n (UAS-*dicer*/201Y; UAS-*dbt*^RNAi^/+) = 38. (C) Average sleep bout duration during the daytime and nighttime with down-regulated expression of *dbt* with mushroom body driver 201Y. n (UAS-*dicer*/+; UAS-*dbt*^RNAi^/+) = 46, n (201Y/+) = 45, n (UAS-*dicer*/201Y; UAS-*dbt*^RNAi^/+) = 38. (D) Activity counts/awake time during the daytime and nighttime with down-regulated expression of *dbt* with mushroom body driver 201Y. n (UAS-*dicer*/+; UAS-*dbt*^RNAi^/+) = 46, n (201Y/+) = 45, n (UAS-*dicer*/201Y; UAS-*dbt*^RNAi^/+) = 38. (E) Loss of expression of *dbt* in α and β lobe of mushroom body (C309) of flies causes a sleep increase during the daytime. n (UAS-*dicer*/+; UAS-*dbt*^RNAi^/+) = 43, n (C309/+) = 43, n (UAS-*dicer*/C309; UAS-*dbt*^RNAi^/+) = 44. (F) Average sleep amount during the daytime and nighttime with down-regulation of *dbt* expression with mushroom body driver C309. n (UAS-*dicer*/+; UAS-*dbt*^RNAi^/+) = 43, n (C309/+) = 43, n (UAS-*dicer*/C309; UAS-*dbt*^RNAi^/+) = 44. (G) Average sleep bout duration during the daytime and nighttime with down-regulated expression of *dbt* with mushroom body driver C309. n (UAS-*dicer*/+; UAS-*dbt*^RNAi^/+) = 43, n (C309/+) = 43, n (UAS-*dicer*/C309; UAS-*dbt*^RNAi^/+) = 44. (H) Activity counts/awake time during the daytime and nighttime with down-regulated expression of *dbt* with mushroom body driver C309. n (UAS-*dicer*/+; UAS-*dbt*^RNAi^/+) = 43, n (C309/+) = 43, n (UAS-*dicer*/C309; UAS-*dbt*^RNAi^/+) = 44. (I) Loss of expression of *dbt* in fan-shaped body (C205) leads to a sleep increase during the daytime. n (UAS-*dicer*/+; UAS-*dbt*^RNAi^/+) = 45, n (C205/+) = 47, n (UAS-*dicer*/+; UAS-*dbt*^RNAi^/C205) = 44. (J) Average sleep amount during the daytime and nighttime with down-regulated expression of *dbt* with fan-shaped body driver C205. n (UAS-*dicer*/+; UAS-*dbt*^RNAi^/+) = 45, n (C205/+) = 47, n (UAS-*dicer*/+; UAS-*dbt*^RNAi^/C205) = 44. (K) Average sleep bout duration during the daytime and nighttime with down-regulated expression of *dbt* with fan-shaped body driver C205. n (UAS-*dicer*/+; UAS-*dbt*^RNAi^/+) = 45, n (C205/+) = 47, n (UAS-*dicer*/+; UAS-*dbt*^RNAi^/C205) = 44. (L) Activity counts/awake time during the daytime and nighttime with down-regulated expression of *dbt* with fan-shaped body driver C205. n (UAS-*dicer*/+; UAS-*dbt*^RNAi^/+) = 45, n (C205/+) = 47, n (UAS-*dicer*/+; UAS-*dbt*^RNAi^/C205) = 44. (M) Loss of expression of *dbt* in ellipsoid body (C819) leads to a sleep increase during the daytime. n (UAS-*dicer*/+; UAS-*dbt*^RNAi^/+) = 46, n (C819/+) = 47, n (UAS-*dicer*/+; UAS-*dbt*^RNAi^/C819) = 46. (N) Average sleep amount during the daytime and nighttime with down-regulated expression of *dbt* with ellipsoid body driver C819. n (UAS-*dicer*/+; UAS-*dbt*^RNAi^/+) = 46, n (C819/+) = 47, n (UAS-*dicer*/+; UAS-*dbt*^RNAi^/C819) = 46. (O) Average sleep bout duration during the daytime and nighttime with down-regulated expression of *dbt* with ellipsoid body driver C819. n (UAS-*dicer*/+; UAS-*dbt*^RNAi^/+) = 46, n (C819/+) = 47, n (UAS-*dicer*/+; UAS-*dbt*^RNAi^/C819) = 46. (P) Activity counts/awake time during the daytime and nighttime with down-regulated expression of *dbt* with ellipsoid body driver C819. n (UAS-*dicer*/+; UAS-*dbt*^RNAi^/+) = 46, n (C819/+) = 47, n (UAS-*dicer*/+; UAS-*dbt*^RNAi^/C819) = 46. (Q) Sleep profile of flies with lower expression of *dbt* from 201Y without expression of PER protein. n (*per*^0^/Y; UAS-*dicer*/+; UAS-*dbt*^RNAi^/+) = 48, n (*per*^0^/Y;201Y/+) = 44, n (*per*^0^/Y; UAS-*dicer*/+; UAS-*dbt*^RNAi^/+) = 44. (R) Average sleep amounts of flies with lower expression of *dbt* from 201Y without expression of PER protein. n (*per*^0^/Y; UAS-*dicer*/+; UAS-*dbt*^RNAi^/+) = 48, n (*per*^0^/Y;201Y/+) = 44, n (*per*^0^/Y; UAS-*dicer*/+; UAS-*dbt*^RNAi^/+) = 44. (S) Sleep profile of flies with lower expression of *dbt* from C309 without expression of PER protein. n (*per*^0^/Y; UAS-*dicer*/+; UAS-*dbt*^RNAi^/+) = 48, n (*per*^0^/Y;C309/+) = 48, n (*per*^0^/Y; UAS-*dicer*/C309; UAS-*dbt*^RNAi^/+) = 47. (T) Average sleep amounts of flies with lower expression of *dbt* from C309 without expression of PER protein. n (*per*^0^/Y; UAS-*dicer*/+; UAS-*dbt*^RNAi^/+) = 48, n (*per*^0^/Y;C309/+) = 48, n (*per*^0^/Y; UAS-*dicer*/C309; UAS-*dbt*^RNAi^/+) = 47. (U) Sleep profile of flies with lower expression of *dbt* from C205 without expression of PER protein. (*per*^0^/Y; UAS-*dicer*/+; UAS-*dbt*^RNAi^/+) = 48, n (*per*^0^/Y;C205/+) = 47, n (*per*^0^/Y; UAS-*dicer*/+; UAS-*dbt*^RNAi^/C205) = 46. (V) Average sleep amounts of flies with lower expression of *dbt* from C205 without expression of PER protein. (*per*^0^/Y; UAS-*dicer*/+; UAS-*dbt*^RNAi^/+) = 48, n (*per*^0^/Y;C205/+) = 47, n (*per*^0^/Y; UAS-*dicer*/+; UAS-*dbt*^RNAi^/C205) = 46. (W) Sleep profile of flies with lower expression of *dbt* from C819 without expression of PER protein. n (*per*^0^/Y; UAS-*dicer*/+; UAS-*dbt*^RNAi^/+) = 48, n (*per*^0^/Y;C819+) = 31, n (*per*^0^/Y; UAS-*dicer*/+; UAS-*dbt*^RNAi^/C819) = 41. (X) Average sleep amounts of flies with lower expression of *dbt* from C819 without expression of PER protein. n (*per*^0^/Y; UAS-*dicer*/+; UAS-*dbt*^RNAi^/+) = 48, n (*per*^0^/Y;C819+) = 31, n (*per*^0^/Y; UAS-*dicer*/+; UAS-*dbt*^RNAi^/C819) = 41. ns. no significant difference, *p< 0.05, **p< 0.01 and ***p< 0.001. Black and grey bars (or lines) respectively represent UAS-/+, *-*GAL4/+ control flies, while orange bars (or lines) represent the treatment flies. The horizontal bar below each graph presents day (white) and night (black).

Previous reports showed that central complex and mushroom body neurons are critical in sleep regulation [2,23,24]. To determine if *dbt* affects sleep through other sites of expression in the central nervous system outside of the circadian neurons and minimize the nonspecific expression of specific drivers, we used various GAL4 lines expressed in the mushroom body (C309, 201Y), fan-shaped body (C205, 23E10-GAL4), ellipsoid body (C232, C819, 44409), and PI (*dilp2*-GAL4) to down-regulate the *dbt* gene endogenously with a UAS-*dicer*; UAS-*dbt*^RNAi^ line; the expression of these drivers are shown in S4 Fig. We found that reduction of expression of *dbt* in the mushroom body (Fig 7A–7H), fan-shaped body (Fig 7I–7L) or ellipsoid body (Fig 7M–7P) causes a sleep increase during daytime but does not affect circadian period, in contrast with the effect obtained with *tim*-GAL4 (Table 1), the data for the other drivers, including 44409, C232, 23E10-GAL4 and *dilp2*-GAL4, that did not cause strong effects are shown in S3 Fig. In addition, according to the analysis of activity counts/awake time, we find that the increased daytime sleep phenotype, is not caused by abnormal activity in these flies (Fig 7D, 7H, 7L and 7P). The daytime sleep in individual *dbt*-GAL4/UAS-*dbt*^RNAi^ flies with both a long circadian rhythm and arrhythmia have also been analyzed respectively, and the results show that the daytime sleep is increased in both of these flies, meaning the sleep effect of *dbt* during daytime is independent of the circadian rhythm phenotype (S6 Fig).

The localization result of *per*-GAL4 shows that this *per* driver is widely expressed in the fly brain, including in clock neurons, EB, AL and the lateral horn (Fig 5M–5R). The previous reports showed that this driver is also expressed in parts of the central complex neurons in addition to clock neurons [22,25], and *dbt* has a similar expression pattern as this driver (Fig 3E and 3F). In addition, as DBT has a close relationship with PER in the circadian program, which is important in sleep regulation, there is a possibility that *dbt* affects sleep in both circadian and non-circadian neurons that are both dependent on PER. Therefore, to investigate if *dbt* effects on sleep through the mushroom body and central complex neurons are dependent on PER protein, we decreased the expression of *dbt* with 201Y (Fig 7Q and 7R), C309 (Fig 7S and 7T), C205 (Fig 7U and 7V) and C819 (Fig 7W and 7X) in a *per*^0^ mutant background. Without PER, the sleep increase during the daytime could not be detected with decreased expression of *dbt* in the mushroom body neurons and central complex neurons. Thus, the effect of *dbt* on sleep is dependent on PER. Although we cannot determine whether PER and DBT are expressed in the same neurons to mediate the daytime increase, at least DBT regulates sleep through PER.

Therefore, decreased expression of *dbt* in the mushroom body, fan-shaped body and ellipsoid body causes an increased sleep amount during daytime, while decreased expression in circadian neurons causes a sleep decrease during nighttime, thereby confirming the possibility that *dbt* affects sleep through circadian neurons (decreased sleep at night), mushroom body and central complex neurons (increased sleep during the day, as does *per-*GAL4 expression).

Furthermore, we knocked down the expression of *dbt* only in adult flies driven by the drivers of the mushroom body, fan-shaped body and ellipsoid body, in conjunction with the *tub*-GAL80ts repressor. The results showed that down-regulation of *dbt* only in the adult stage did not impact sleep during the daytime, indicating that effects of *dbt* on sleep in these neurons is dependent on development (S7 Fig).

As we have mentioned above, decreased expression of *dbt* with *dbt*-GAL4 causes a sleep increase during the daytime and a sleep decrease during the nighttime. Decreased expression of *dbt* in clock neurons causes a sleep decrease during the nighttime while the daytime sleep does not change. Finally, decreased expression of *dbt* in the mushroom body or central complex neurons only causes a sleep increase during the daytime while the nighttime sleep does not change. Therefore, reduced expression with *dbt*-GAL4 generates both the effects produced by knock-down in circadian and noncircadian neurons, presumably because expression is reduced in both types of neurons.

## Discussion

In circadian rhythms, DBT is an important kinase that can degrade PER during the day, with PER stabilization in a binary complex with TIM at night and eventual movement of the PER/TIM complex into the nucleus around midnight [8,26]. Due to its multiple functions in the circadian clock, loss of function of *dbt* may cause different circadian mutations such as short period, long period and arrhythmic flies. There are several reports showing that the human sleep disorder Familial Advanced Sleep Phase Syndrome (FASPS) could be explained as mutations of phosphorylation sites in PER or mutations of CKIε or δ (human orthologues of *dbt*) [17,27], thereby indicating a function of *dbt* on clock-dependent sleep regulation. We monitored sleep in two different *dbt* mutants. Both mutants show a sleep decrease during the nighttime, but the difference is that in the *dbt*^S^ mutant, the sleep decrease happens in the late evening while in the *dbt*^L^ mutant, it happens in the early morning and the whole night. The timing of these phenotypes suggests that the phase change of locomotor activity may be the main reason why these different *dbt* mutants affect sleep differently.

As different sleep phenotypes were seen in the two mutants, the sleep phenotype produced by loss of expression for wild type *dbt* was assessed. There is a report showing that *dbt* is expressed widely in the optic lobes, clock neurons including in the lateral neurons, and has the same pattern as *per* transcription in the adult head [7]. In order to investigate the localization of *dbt* and to down-regulate the expression of *dbt* endogenously, a *dbt*-GAL4 line was constructed. Then we used a UAS-mCD8: GFP reporter expressed with *dbt*-GAL4 to observe the expression sites of *dbt*. The localization of *dbt*-GAL4 expression pattern in our study shows that *dbt* is not only expressed in clock neurons but is also broadly expressed in the mushroom body and central complex neurons. To specifically assess the function of all these neurons in the regulation of sleep by *dbt*, a UAS-*dbt*^RNAi^ line was expressed with the *dbt*-GAL4 driver to decrease the expression of *dbt* in all of these areas. This knock-down produced two *dbt* phenotypes–elevated daytime sleep and reduced nighttime sleep.

In order to rule out developmental effects, we added a *tub*-GAL80ts to inhibit GAL4 mediated expression of *dbt* RNAi during development and then down-regulated the expression of *dbt* for 3–5 days after the adults emerged from the pupae. The sleep assay results show that the effect of *dbt* on daytime sleep is likely to be based on developmental processes, while the sleep reduction during the nighttime is due to the loss of expression of *dbt* in the adult stage. Thus, we propose that the *dbt* kinase may affect sleep through different pathways that depend on different groups of neurons.

As it is known that *dbt* is expressed in clock neurons, the *tim-*GAL4 reported to be mainly expressed in clock neurons and in some glial cells was used to down-regulate *dbt* [22]; the flies showed a sleep reduction during the nighttime. As use of a glial GAL4 drivers to down regulate *dbt* did not cause any significant changes in sleep, the effect of knocking down *dbt* with *tim*-GAL4 on sleep is likely due to clock neurons rather than glial cells and central complex neurons. Moreover, the decreased sleep amount during the nighttime is consistent with the sleep phenotype of the *dbt* mutants. As the RNAi-system has the possibility of off-target effects on other mRNAs, the UAS-*dbt*^K/R^ line was used here as another *dbt* loss-of-function methodology. K38 and the surrounding region are conserved in *Drosophila dbt* and vertebrate CKIε/δ, and the mutation can cause a longer circadian period or arrhythmicity in a dominant negative manner [11]. A previous study shows that with altered kinase activity a mutant of CKIε/δ may cause sleep advance or delay in humans [17,28], which is the same phenotype produced in *Drosophila*. For example, sleep is advanced in the short period *dbt*^S^ mutant and delayed in the longer period *dbt*^L^ flies. Although various sleep phenotypes may happen in *dbt* mutants, none of them show the unique sleep phenotype that *dbt*^K/R^ shows. The sleep analysis of UAS-*dbt*^K/R^/*tim-*GAL4 indicates that the *dbt*^K/R^ mutant can cause either sleep loss during the nighttime or a sleep decrease every other night, and this sleep profile is dependent on PER protein. It is also possible that this alternating sleep profile is caused by a longer period (~30 h) in the UAS-*dbt*^K/R^/*tim*-GAL4 line–a period that persists even in LD because it cannot entrain to the 24 hr LD cycle. While two different sleep phenotypes were shown with this genotype, the sleep amount during the nighttime is still decreased in both groups in UAS-*dbt*^K/R^/*tim-*GAL4 flies, and this result is consistent with the result for knock-down with UAS-*dbt*^RNAi^ and the *tim*-GAL4 driver. These results show that down-regulation of *dbt* levels or overexpression of a dominant negative *dbt* mutant in clock neurons can both cause a sleep decrease, which is dependent on PER because the effect is eliminated in a *per*^*0*^ mutant.

With down-regulation of *dbt* in clock neurons and *dbt*-GAL4 neurons, the flies exhibit a sleep decrease during the nighttime. When the *per-*GAL4 driver (another clock neuron driver) was used to reduce the expression of *dbt* transcription, the flies showed a sleep increase during the daytime only. The expression patterns of the *tim*-GAL4 and *per*-GAL4 drivers have been described [22] as different. The *per-*GAL4 is not only expressed in clock neurons but also in the central brain. That might be the main reason why the UAS-*dbt*^RNAi^/*per-*GAL4 flies have a higher daytime sleep. However, the expression pattern of the *per*-GAL4 driver in clock neurons is not as extensive as the *tim*-GAL4 and *dbt*-GAL4 drivers. That is, the *per*-GAL4 used in this study is expressed only in 2–3 cells of the l-LNv (Fig 5N) and DN1s (Fig 5O), while *tim*-Gal4 and *dbt*-Gal4 are broadly expressed in clock cells such as the LNvs (Figs 3I, 3J, and 5I–5L). As *per-*GAL4 is also expressed in clock neurons, it should have the potential to reduce sleep during the nighttime, but this has not been detected. It is possible that down-regulation of *dbt* with the *per-*GAL4 driver is not strong enough to cause a circadian effect, so that sleep reduction during the nighttime may not be observed. In support of this, the *per-*GAL4 driver did not lengthen circadian period as did *tim-*GAL4 and *dbt*-GAL4 during DD (Table 1). These results mean that the circadian clock may not be the only source of sleep change in *dbt* mutants.

Various studies have shown that the central complex neurons and mushroom body are crucial to the regulation of sleep [2]. For instance, the activation or silencing of the mushroom body may promote or reduce sleep of flies, showing that the mushroom body plays an important role in sleep regulation [24,29]. Additionally, the fan-shaped body and ellipsoid body have been reported to contribute to sleep/wake promotion and homeostasis regulation [23,30]. In order to localize the site of mediation by *dbt* of the developmental effect on daytime sleep, we down-regulated *dbt* in several central complex and mushroom body neurons with different GAL4 drivers, and the results indicated that down-regulation of *dbt* in these neurons resulted in an increased daytime sleep, which is consistent with the effect caused by the *per-*GAL4 driver. In conclusion, *dbt* may affect sleep through both circadian neurons and noncircadian neurons. As we have mentioned above, the sleep decrease during the nighttime in UAS-*dbt*^K/R^/*tim-*GAL4 flies is dependent on PER, and the localization of *dbt* and *per* drivers shown in this paper indicates that both of the genes may be expressed in the central complex neurons and some part of the mushroom body neurons. Previous reports also showed that *per*-GAL4 is expressed broadly in the central nervous system [22,25], and PER is expressed in more neurons after knocking down DBT [8]. Therefore, we determined if *dbt* effects on daytime sleep are also dependent on PER. To investigate this hypothesis, we decreased the expression of DBT with various GAL4 lines in a *per*^0^ background, with the disappearance of the daytime sleep increase in the treatment flies. This result shows that *dbt* effects on daytime sleep are also dependent on PER. The requirement of PER for both the DBT-dependent increase in daytime sleep and decrease in nighttime sleep indicates that these effects are likely central to the sleep mechanism rather than indirect effects of DBT on sleep. Which developmental pathway contributes to *dbt’s* effects on sleep regulation, and how do the circadian and noncircadian cells produce different effects on sleep? Additional work will address these issues.

## Materials and methods

### Fly strains

The UAS-*dbt*^RNAi^ (VDRC: v9241) line was from the Vienna *Drosophila* RNAi center; the *dbt* mutants, including *dbt*^S^, *dbt*^L^ and the UAS-*dbt*^K/R^ line were previously described [8,11,31]; the UAS-*dicer* (I); *tim-*GAL4 line was previously described [32]; the UAS-*dicer* (II) (BDSC: 24650), *dilp2*-GAL4 (BDSC: 37516), 23E10-GAL4 (BDSC: 49032), EB-GAL4(BDSC: 44409), Cre;D/Sb (BDSC: 851) and *tub*-GAL80ts (BDSC: 7108) were from Bloomington Stock Center; the *tim-*GAL4 (BDSC: 7126) and *per-*GAL4 (BDSC: 7127) drivers were previously reported [22,33]; the *repo*-GAL4 was provided by Dr. Yi Rao (Peking University), the C309 [24,34,35] and 201Y [24,36] lines were provided by Dr. Yan Li (Institute of Biophysics, Chinese Academy of Science); UAS-CD8: mGFP (BDSC: 5137) [37], C205 [38], 44409 [39], C232 and C819 [40] were provided by Dr. Li Liu (Institute of Biophysics, Chinese Academy of Science). The mutants *dbt*^S^ and *dbt*^L^ have been backcrossed with Canton-S for 6 generations and all experiments have been repeated at least 3 times in the manuscript.

### Generation of *dbt*-GAL4 line with a Crispr-Cas 9 approach

The *dbt*-GAL4 line was constructed with the Crispr-Cas 9 approach by inserting the T2AGAL4 reading frame fused in frame immediately downstream of the last amino acid in *dbt*. The donor, which has *dbt* regions extending from 3R:31055021 to 31058029 with a T2A-GAL4 sequence and a removable marker LoxP-RFP-LoxP downstream of the last amino acid of *GAL4*, was carried in the pBluescript-SK (+) vector.

The sgRNAs were designed with CRISPR Optimal Target Finder (http://tools.flycrispr.molbio.wisc.edu/targetFinder/) [41]. With the goal of enhanced cleavage of the target site, they were designed as follows:

gRNA1 (genomic target sequence + gRNA scaffold sequence):

GCAAATAATATTTTATCGTTT+GTTTTAGAGCTAGAAATAGCAAGTTAAAATAAGGCTAGTCCGTTATCAACTTGAAAAAGTGGCACCGAGTCGGTGC

gRNA2 (genomic target sequence + gRNA scaffold sequence):

GTATCGTTTAGGTTGCGACGC+GTTTTAGAGCTAGAAATAGCAAGTTAAAATAAGGCTAGTCCGTTATCAACTTGAAAAAGTGGCACCGAGTCGGTGC;

gRNA3 (genomic target sequence + gRNA scaffold sequence):

GTTGAATGTATCCAAGCGGC+GTTTTAGAGCTAGAAATAGCAAGTTAAAATAAGGCTAGTCCGTTATCAACTTGAAAAAGTGGCACCGAGTCGGTGC

Cas9 mRNA was produced following the protocol of Basset et al. [42].

The construction of donor, RNA mix injection and screening of transgenic flies were performed by Fungene Biotech (http://www.fungene.tech/). The G0 flies were crossed with w^1118^; TM3, Ser/TM6, Tb line, and the positive flies were identified by the RFP marker which is expressed in the eyes and eventually removed by crossing with the Cre; D/Sb (line 851 from Bloomington Drosophila Stock Center).

### Behavioral assays

3–5 days old flies were placed in 65mm×5mm glass tubes with 0.54% agar, 1.1% yeast, 5.6% corn powder, 6.8% molasses and 0.1% Tegosept fly food. Sleep assays were performed at ~25°C, ~60% humidity, 12h light/12h dark (LD) conditions, and for circadian assays, the flies were entrained in LD for 3 days and detected in constant darkness (DD) for 5–7 days. Locomotor activity was detected using the DAM system (Drosophila activity monitoring system, Trikinetics, Waltham, MA), and periods of inactivity lasting 5-min or more were quantified as sleep as previously described. Flies in experiments with *tub*-GAL80ts were raised and analyzed at either 18°C, 22°C or 29°C as specified in Results. The data of sleep analysis is following the method of Driscoll et al [43] with Matlab and pysolo [44]. Circadian activity assays and analysis were conducted by faasX software.

### Immunofluorescence assays

The brains of adult flies were dissected in chilled phosphate buffer saline (PBS) and fixed in 4% paraformaldehyde in PBS (PFA) for 1–2 h, and then washed in washing buffer (1% Triton-X 100 in PBS, or PBST1). After 3 washes of 10 minutes each, the brains were blocked with PBTN (10% goat serum and 0.5% Triton-X 100 in PBS) for at least 2 h and then incubated overnight with primary antibodies. The following primary antibodies were used: mouse anti PDF (Cat#: PDF C7; RRID: AB_760350; dilute rate: 1:1000), mouse anti GFP (CAT#: DSHB-GFP-12A6; RRID: AB_2617417; dilute rate: 1:200) and mouse anti NC82 (Cat#: nc82; RRID: AB_2314866; dilute rate: 1:200) from DHSB, rabbit anti GFP (Cat#: G10362; RRID: AB_2536526; dilute rate: 1:1000) from Invitrogen, and rabbit anti PER (1:10000) from the Price lab [11]. After incubation with these primary antibodies, brains were washed in washing buffer 2 (0.5% Triton in PBS, PBST2) 3 times and were incubated with secondary antibodies for 2h. The secondary antibodies used were goat anti mouse CY5 (Cat#: 115-175-146; RRID: AB_2338713; dilute rate: 1:200) and goat anti rabbit CY5 (Cat#: 111-175-144; RRID: AB_2338013; dilute rate: 1:200) from Jackson immune Research, goat anti rabbit 488 (Cat#: A-31565; RRID: AB_2536178; dilute rate: 1:1000) from Invitrogen, and goat anti mouse FITC (Cat#: ZF-0312; dilute rate: 1:200) from zsbio, Beijing, China. The fluorescently labeled brains were scanned by Leica SP8 confocal, Olympus Fluoview confocal and Zeiss LSM800 confocal microscopy.

### Statistical analysis

All the sleep profiles and the significance analyses are produced by GraphPad Prism 8.0. All the parameters of statistical analysis are shown in S2–S12 Tables.

### Resource ability

#### Materials availability

Fly lines and antibodies not available commercially can be obtained from one of the three labs involved in this study (Zhao, Price or Dissel).

## Supporting information

S1 Fig Sleep phenotype of female flies with mutant *dbt* and lower expression of *dbt*. (A) Sleep profiles of *dbt*^S^ female flies. n (CS) = 45, n (*dbt*^S^) = 39. (B) Average sleep amount during the daytime and nighttime in *dbt*^S^ female flies. (daytime: t = 5.556, df = 82, ***p <0.0001 by unpaired t-test; nighttime: t = 9.431, df = 82, ***p <0.0001 by unpaired t-test). n (CS) = 45, n (*dbt*^S^) = 39. (C) Sleep profiles of *dbt*^L^ female flies. n (CS) = 48, n (*dbt*^L^) = 44. (D) Average sleep amount during the daytime and nighttime in *dbt*^L^ female flies (daytime: t = 5.945, df = 90, ***p<0.0001 by unpaired t-test; nighttime: t = 3.426, df = 90, ***p = 0.0009 by unpaired t-test). n (CS) = 48, n (*dbt*^L^) = 44. (E) Sleep profile of female flies with down-regulated *dbt* expression from *tim-*GAL4. n (UAS-*dbt*^RNAi^/+) = 42, n (UAS-*dicer*/+; *tim*-GAL4/+) = 47, n (UAS-*dicer*/+; *tim*-GAL4/+; UAS-*dbt*^RNAi^/+) = 47. (F) Average sleep amount during daytime and nighttime with down-regulated *dbt* expression from *tim-*GAL4 driver. n (UAS-*dbt*^RNAi^/+) = 42, n (UAS-*dicer*/+; *tim*-GAL4/+) = 47, n (UAS-*dicer*/+; *tim*-GAL4/+; UAS-*dbt*^RNAi^/+) = 47. (G) Sleep profile of female flies with down-regulated *dbt* expression from *per-*GAL4. n (UAS-*dicer*/+; UAS-*dbt*^RNAi^/+) = 46, n (*per*-GAL4/+) = 45, n (UAS-*dicer*/ *per*-GAL4; UAS-*dbt*^RNAi^/+) = 45. (H) Average sleep amount during daytime and nighttime with down-regulated *dbt* expression from *per-*GAL4 driver. n (UAS-*dicer*/+; UAS-*dbt*^RNAi^/+) = 46, n (*per*-GAL4/+) = 45, n (UAS-*dicer*/ *per*-GAL4; UAS-*dbt*^RNAi^/+) = 45. (I) Loss of expression of *dbt* in α, β and γ lobes of mushroom body (201Y) of flies cause a sleep increase during the daytime. n (UAS-*dicer*/+; UAS-*dbt*^RNAi^/+) = 47, n (201Y/+) = 48, n (UAS-*dicer*/201Y; UAS-*dbt*^RNAi^/+) = 38. (J) Average sleep amount during the daytime and nighttime with down-regulation of *dbt* expression with mushroom body driver 201Y. n (UAS-*dicer*/+; UAS-*dbt*^RNAi^/+) = 47, n (201Y/+) = 48, n (UAS-*dicer*/201Y; UAS-*dbt*^RNAi^/+) = 38. (K) Loss of expression of *dbt* in α and β lobe of mushroom body (C309) of flies causes a sleep increase during the daytime. n (UAS-*dicer*/+; UAS-*dbt*^RNAi^/+) = 42, n (C309/+) = 41, n (UAS-*dicer*/C309; UAS-*dbt*^RNAi^/+) = 44. (L) Average sleep amount during the daytime and nighttime with down-regulation of *dbt* expression with mushroom body driver C309. n (UAS-*dicer*/+; UAS-*dbt*^RNAi^/+) = 42, n (C309/+) = 41, n (UAS-*dicer*/C309; UAS-*dbt*^RNAi^/+) = 44. (M) Loss of expression of *dbt* in fan-shaped body (C205) leads to a sleep increase during the daytime. n (UAS-*dicer*/+; UAS-*dbt*^RNAi^/+) = 47, n (C205/+) = 46, n (UAS-*dicer*/+; UAS-*dbt*^RNAi^/C205) = 44. (N) Average sleep amount during the daytime and nighttime with down-regulated expression of *dbt* with fan-shaped body driver C205. n (UAS-*dicer*/+; UAS-*dbt*^RNAi^/+) = 47, n (C205/+) = 46, n (UAS-*dicer*/+; UAS-*dbt*^RNAi^/C205) = 44. (O) Loss of expression of *dbt* in ellipsoid body (C819) leads to a sleep increase during the daytime. n (UAS-*dicer*/+; UAS-*dbt*^RNAi^/+) = 41, n (C819/+) = 48, n (UAS-*dicer*/+; UAS-*dbt*^RNAi^/C819) = 42. (P) Average sleep amount during the daytime and nighttime with down-regulated expression of *dbt* with ellipsoid body driver C819. n (UAS-*dicer*/+; UAS-*dbt*^RNAi^/+) = 41, n (C819/+) = 48, n (UAS-*dicer*/+; UAS-*dbt*^RNAi^/C819) = 42. ns. no significant difference, *p< 0.05, **p< 0.01 and ***p< 0.001. Black and purple bars (or lines) respectively represent data of wildtype and *dbt* mutant in A-D, in E-P, Black and grey bars (or lines) respectively represent UAS-/+, *-*GAL4/+ control flies, while purple bars (or lines) represent the treatment flies. The horizontal bar below each graph presents day (white) and night (black).
(TIF)

S2 Fig Sleep phenotypes of flies with higher expression of *dbt*^K/R^ mutant only during adulthood. (A) Sleep profile of flies with adult specific expression of *dbt*^K/R^. The flies were raised at 18°C and sleep was detected at 22°C for 1 day (blue background) and 29°C for 2 days (pink background). The sleep parameters of the day which is marked with red-dotted frame is shown in B-E. n (UAS-*dbt*^K/R^*/+*) = 47, n (*tub-*GAL80ts*/+; dbt*-GAL4/+) = 46, n (*tub-GAL80ts/+*; UAS-*dbt*^K/R^/*dbt*-GAL4) = 46. (B) Total sleep of flies with higher expression of *dbt*^K/R^ in adult stage. The total sleep was significantly decreased during the nighttime with no significant difference during the daytime. n (UAS-*dbt*^K/R^/+) = 47, n (*tub*-GAL80ts/+; *dbt*-GAL4/+) = 46, n (*tub*-GAL80ts/+; UAS-*dbt*^K/R^/*dbt*-GAL4) = 46. (C) Sleep bout duration of flies with higher expression of *dbt*^K/R^ in adult stage. n (UAS-*dbt*^K/R^/+) = 47, n(*tub*-GAL80ts/+; *dbt*-GAL4/+) = 46, n (*tub*-GAL80ts/+; UAS-*dbt*^K/R^/*dbt*-GAL4) = 46. (D) Activity counts/awake time of flies with higher expression of *dbt*^K/R^ in adult stage. n (UAS-*dbt*^K/R^/+) = 47, n (*tub*-GAL80ts/+; *dbt*-GAL4/+) = 46, n (*tub*-GAL80ts/+; UAS-*dbt*^K/R^/*dbt*-GAL4) = 46. ns. no significant difference, *p< 0.05, **p< 0.01 and ***p< 0.001. Black and grey bars (or lines) respectively represent UAS-/+, -GAL4/+ control flies, while orange bars (or lines) represent the treatment flies. The horizontal bar below each graph presents day (white) and night (black).
(TIF)

S3 Fig Knock down of *dbt* expression in some parts of the central neuron system, did not cause a strong effect on sleep. (A) Loss of expression of *dbt* with ellipsoid body driver (44409) of flies causes a slight sleep increase during the daytime. n (UAS-*dicer*/+; UAS-*dbt*^RNAi^/+) = 46, n (44409/+) = 47, n (UAS-*dicer*/+; UAS-*dbt*^RNAi^/44409) = 46. (B) Total sleep of flies with lower *dbt* from ellipsoid body 44409. n (UAS-*dicer*/+; UAS-*dbt*^RNAi^/+) = 46, n (44409/+) = 47, n (UAS-*dicer*/+; UAS-*dbt*^RNAi^/44409) = 46. (C) Sleep profiles of flies with down-regulation of *dbt* expression with ellipsoid body driver C232. n (UAS-*dicer*/+; UAS-*dbt*^RNAi^/+) = 48, n (C232/+) = 45, n (UAS-*dicer*/+; UAS-*dbt*^RNAi^/C232) = 46. (D) Total sleep of flies with lower *dbt* with C232. n (UAS-*dicer*/+; UAS-*dbt*^RNAi^/+) = 48, n (C232/+) = 45, n (UAS-*dicer*/+; UAS-*dbt*^RNAi^/C232) = 46. (E) Loss of expression of *dbt* in dorsal fan-shaped body (23E10-GAL4) of flies did not show an effect on sleep. n (UAS-*dicer*/+; UAS-*dbt*^RNAi^/+) = 44, n (23E10-GAL4/+) = 40, n (UAS-*dicer*/+; UAS-*dbt*^RNAi^/23E10-GAL4) = 45. (F) Total sleep of flies with lower *dbt* in dorsal fan-shaped body. n (UAS-*dicer*/+; UAS-*dbt*^RNAi^/+) = 44, n (23E10-GAL4/+) = 40, n (UAS-*dicer*/+; UAS-*dbt*^RNAi^/23E10-GAL4) = 45. (G) Loss expression of *dbt* in pars intercerebralis (*dilp2*-GAL4) of flies did not show an effect on sleep. n (UAS-*dicer*/+; UAS-*dbt*^RNAi^/+) = 45, n (*dilp2*-GAL4/+) = 45, n (UAS-*dicer*/*dilp2*-GAL4; UAS-*dbt*^RNAi^/+) = 48. (H) Total sleep of flies with lower *dbt* in pars intercerebralis. n (UAS-*dicer*/+; UAS-*dbt*^RNAi^/+) = 45, n (*dilp2*-GAL4/+) = 45, n (UAS-*dicer*/*dilp2*-GAL4; UAS-*dbt*^RNAi^/+) =. (I) Loss expression of *dbt* in glial cells (*repo*-GAL4) of flies did not show an effect on sleep. n (UAS-*dicer*/+; UAS-*dbt*^RNAi^/+) = 48, n (*repo*-GAL4/+) = 48, n (UAS-*dicer/+*; UAS-*dbt*^RNAi^/ *repo*-GAL4) = 42. (J) Total sleep of flies with lower *dbt* in glial cells. n (UAS-*dicer*/+; UAS-*dbt*^RNAi^/+) = 48, n (*repo*-GAL4/+) = 48, n (UAS-*dicer*/*+*; UAS-*dbt*^RNAi^/*repo*-GAL4) = 42. ns. no significant difference, *p< 0.05, **p< 0.01 and ***p< 0.001. Black and grey bars (or lines) respectively represent UAS-/+, *-*GAL4/+ control flies, while orange bars (or lines) represent the treatment flies. The horizontal bar below each graph presents day (white) and night (black).
(TIF)

S4 Fig Expression patterns of various GAL4 lines. (A-I) Expression patterns of drivers detected with UAS-mCD8: GFP and stained with anti-GFP (green) and anti-NC82 (red), from A-I as follows: mushroom body: UAS-mCD8: GFP/201Y, UAS-mCD8: GFP/C309, fan shaped body: UAS-mCD8: GFP/C205, UAS-mCD8: GFP/23E10-GAL4, ellipsoid body: UAS-mCD8: GFP/44409, UAS-mCD8: GFP/C232, UAS-mCD8: GFP/C819, pars intercerebralis: UAS-mCD8: GFP/*dilp2*-GAL4, glia cells: UAS-mCD8: GFP/*repo*-GAL4.
(TIF)

S5 Fig Increased expression of *dbt*^WT^ in clock neurons and some parts of the central brain did not cause a strong effect on sleep. (A) Sleep profiles of flies with higher expression of *dbt*^WT^ with *tim*-GAL4 driver. n (UAS-*dbt*^WT^/+) = 46, n (*tim*-GAL4/+) = 47, (*tim*-GAL4/+; UAS-*dbt*^WT^/+) = 48. (B) Total sleep of flies with higher expression of *dbt*^WT^ with *tim*-GAL4 driver. n (UAS-*dbt*^WT^/+) = 46, n (*tim*-GAL4/+) = 47, (*tim*-GAL4/+; UAS-*dbt*^WT^/+) = 48. (C) Sleep profiles of flies with higher expression of *dbt*^WT^ with *per*-GAL4 driver. n (UAS-*dbt*^WT^/+) = 44, n (*per*-GAL4/+) = 48, (*per-*GAL4/+; UAS-*dbt*^WT^/+) = 46. (D) Total sleep of flies with higher expression of *dbt*^WT^ with *per*-GAL4 driver. n (UAS-*dbt*^WT^/+) = 44, n (*per*-GAL4/+) = 48, (*per-*GAL4/+; UAS-*dbt*^WT^/+) = 46. ns. no significant difference, *p< 0.05, **p< 0.01 and ***p< 0.001. Black and grey bars (or lines) respectively represent UAS-/+, *-*GAL4/+ control flies, while orange bars (or lines) represent the treatment flies. The horizontal bar below each graph presents day (white) and night (black).
(TIF)

S6 Fig *dbt*-regulated daytime sleep is independent of rhythmicity. Both UAS-*dbt*^RNAi^/*dbt*-GAL4 flies with normal and loss of circadian rhythm have a higher daytime sleep than UAS-*dbt*^RNAi^/+ and *dbt*-GAL4/+ flies, indicating that the sleep increased during the daytime in UAS-*dbt*^RNAi^/*dbt*-GAL4 flies independent of the clock. n (UAS-*dbt*^RNAi^/+) = 29, n (*dbt*-GAL4/+) = 31, n (UAS-*dbt*^RNAi^/*dbt*-GAL4) = 15 in rhythmic flies, n (UAS-*dbt*^RNAi^/+) = 5, n (*dbt*-GAL4/+) = 4, n (UAS-*dbt*^RNAi^/*dbt*-GAL4) = 12 in arrhythmic flies. ns. no significant difference, *p< 0.05, **p< 0.01 and ***p< 0.001.
(TIF)

S7 Fig Sleep phenotypes of flies with *dbt* knocking down in mushroom body and central complex body only during adulthood. These flies were raised at 18°C and sleep was detected at 22°C for one day (blue background) and 29°C for 4 days (pink background). The total sleep of days which were marked with red-dotted frame in A, C, E, G and I were shown respectively in B, D, F, H and J. (A) Sleep profile of flies with loss expression of *dbt* in *per*-GAL4 neurons only during adulthood. n (UAS-*dicer*/+; UAS-*dbt*^RNAi^/+) = 52, n (*per*-GAL4/+; *tub*-GAL80ts/+) = 47, n (UAS-*dicer*/*per*-GAL4; UAS-*dbt*^RNAi^/*tub*-GAL80ts) = 41. (B) Total sleep of flies with loss expression of *dbt* in *per*-GAL4 neurons only during adulthood. n (UAS-*dicer*/+; UAS-*dbt*^RNAi^/+) = 52, n (*per*-GAL4/+; *tub*-GAL80ts/+) = 47, n (UAS-*dicer*/*per*-GAL4; UAS-*dbt*^RNAi^/*tub*-GAL80ts) = 41. (C) Sleep profile of flies with loss expression of *dbt* in α, β and γ lobes of mushroom body (201Y) only during adulthood. n (UAS-*dicer*/+; UAS-*dbt*^RNAi^/+) = 52, n (201Y /+; *tub*-GAL80ts/+) = 41, n (UAS-*dicer*/201Y; UAS-*dbt*^RNAi^/*tub*-GAL80ts) = 40. (D) Total sleep of flies with loss expression of *dbt* in α, β and γ lobes of mushroom body (201Y) only during adulthood. n (UAS-*dicer*/+; UAS-*dbt*^RNAi^/+) = 52, n (201Y /+; *tub*-GAL80ts/+) = 41, n (UAS-*dicer*/201Y; UAS-*dbt*^RNAi^/*tub*-GAL80ts) = 40. (E) Sleep profile of flies with loss expression of *dbt* in α and β lobes of mushroom body (C309) only during adulthood. n (UAS-*dicer*/+; UAS-*dbt*^RNAi^/+) = 52, n (C309 /+; *tub*-GAL80ts/+) = 45, n (UAS-*dicer*/C309; UAS-*dbt*^RNAi^/*tub*-GAL80ts) = 36. (F) Total sleep of flies with loss expression of *dbt* in α and β lobes of mushroom body (C309) only during adulthood. n (UAS-*dicer*/+; UAS-*dbt*^RNAi^/+) = 52, n (C309 /+; *tub*-GAL80ts/+) = 45, n (UAS-*dicer*/C309; UAS-*dbt*^RNAi^/*tub*-GAL80ts) = 36. (G) Sleep profile of flies with loss expression of *dbt* in fan-shaped body (C205) only during adulthood. n (UAS-*dicer*/+; UAS-*dbt*^RNAi^/+) = 52, n (*tub*-GAL80ts/+; C205 /+) = 45, n (UAS-*dicer*/ *tub*-GAL80ts; UAS-*dbt*^RNAi^/C205) = 34. (H) Total sleep of flies with loss expression of *dbt* in fan-shaped body (C205) only during adulthood. n (UAS-*dicer*/+; UAS-*dbt*^RNAi^/+) = 52, n (*tub*-GAL80ts/+; C205 /+) = 45, n (UAS-*dicer*/ *tub*-GAL80ts; UAS-*dbt*^RNAi^/C205) = 34. (I) Sleep profile of flies with loss expression of *dbt* in ellipsoid body (C819) only during adulthood. n (UAS-*dicer*/+; UAS-*dbt*^RNAi^/+) = 52, n (*tub*-GAL80ts/+; C819 /+) = 43, n (UAS-*dicer*/ *tub*-GAL80ts; UAS-*dbt*^RNAi^/C819) = 46. (J) Total sleep of flies with loss expression of *dbt* in ellipsoid body (C819) only during adulthood. n (UAS-*dicer*/+; UAS-*dbt*^RNAi^/+) = 52, n (*tub*-GAL80ts/+; C819 /+) = 43, n (UAS-*dicer*/ *tub*-GAL80ts; UAS-*dbt*^RNAi^/C819) = 46. ns. no significant difference, *p< 0.05, **p< 0.01 and ***p< 0.001. Black and grey bars (or lines) respectively represent UAS-/+, -GAL4/+ control flies, while orange bars (or lines) represent the treatment flies. The horizontal bar below each graph presents day (white) and night (black).
(TIF)

S1 Table Pupae number and eclosion rate of flies with lower expression of *dbt* in adult and larval age. (XLSX)

S2 Table Statistic parameters of Fig 3. (XLSX)

S3 Table Statistic parameters of Fig 4. (XLSX)

S4 Table Statistic parameters of Fig 5. (XLSX)

S5 Table Statistic parameters of Fig 6. (XLSX)

S6 Table Statistic parameters of Fig 7. (XLSX)

S7 Table Statistic parameters of S1 Fig. (XLSX)

S8 Table Statistic parameters of S2 Fig. (XLSX)

S9 Table Statistic parameters of S3 Fig. (XLSX)

S10 Table Statistic parameters of S5 Fig. (XLSX)

S11 Table Statistic parameters of S6 Fig. (XLSX)

S12 Table Statistic parameters of S7 Fig. (XLSX)
