# Supplementary figures and images for: DBT affects sleep in both circadian and non-circadian neurons
Source: PLoS Genet. 2022 Feb 9;18(2):e1010035. doi: 10.1371/journal.pgen.1010035 (PMC8827452; doi:10.1371/journal.pgen.1010035)

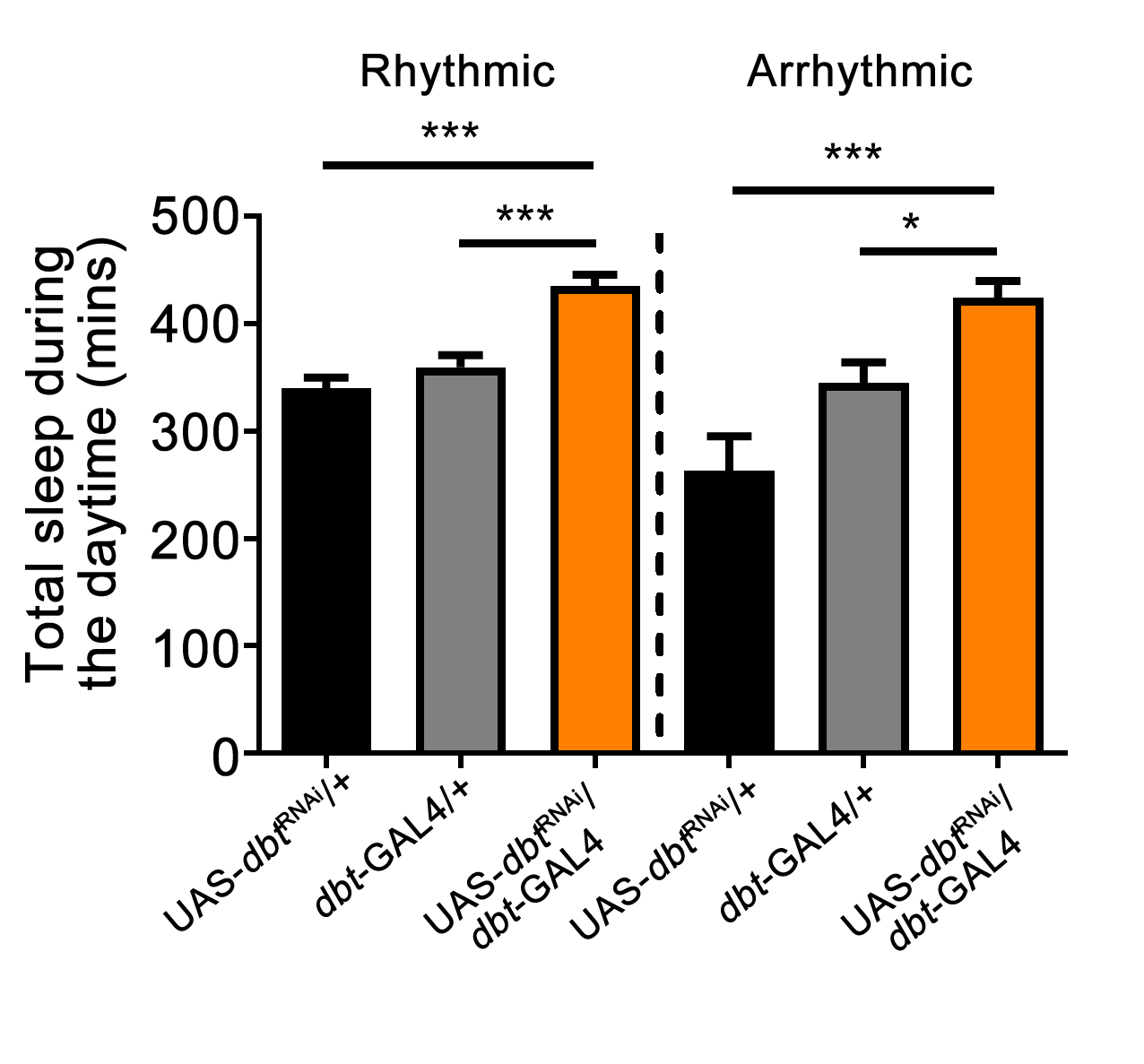

Supplement: S6 Fig — Both UAS-dbtRNAi/dbt-GAL4 flies with normal and loss of circadian rhythm have a higher daytime sleep than UAS-dbtRNAi/+ and dbt-GAL4/+ flies, indicating that the sleep increased during the daytime in UAS-dbtRNAi/dbt-GAL4 flies independent of the clock. n (UAS-dbtRNAi/+) = 29, n (dbt-GAL4/+) = 31, n (UAS-dbtRNAi/dbt-GAL4) = 15 in rhythmic flies, n (UAS-dbtRNAi/+) = 5, n (dbt-GAL4/+) = 4, n (UAS-dbtRNAi/dbt-GAL4) = 12 in arrhythmic flies. ns. no significant difference, *p< 0.05, **p< 0.01 and ***p< 0.001. (TIF) [file pgen.1010035.s006.tif]

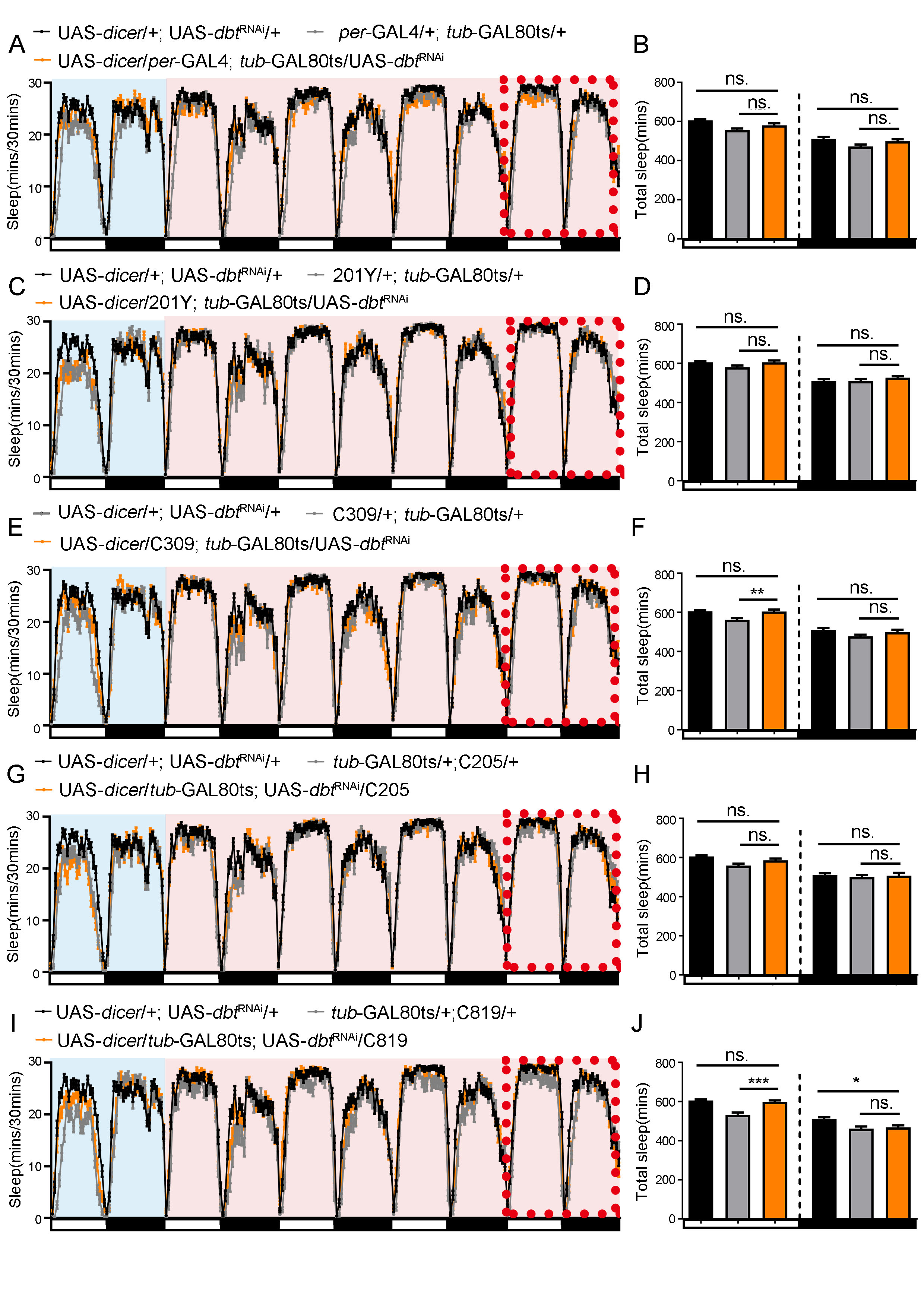

Supplement: S7 Fig — These flies were raised at 18°C and sleep was detected at 22°C for one day (blue background) and 29°C for 4 days (pink background). The total sleep of days which were marked with red-dotted frame in A, C, E, G and I were shown respectively in B, D, F, H and J. (A) Sleep profile of flies with loss expression of dbt in per-GAL4 neurons only during adulthood. n (UAS-dicer/+; UAS-dbtRNAi/+) = 52, n (per-GAL4/+; tub-GAL80ts/+) = 47, n (UAS-dicer/per-GAL4; UAS-dbtRNAi/tub-GAL80ts) = 41. (B) Total sleep of flies with loss expression of dbt in per-GAL4 neurons only during adulthood. n (UAS-dicer/+; UAS-dbtRNAi/+) = 52, n (per-GAL4/+; tub-GAL80ts/+) = 47, n (UAS-dicer/per-GAL4; UAS-dbtRNAi/tub-GAL80ts) = 41. (C) Sleep profile of flies with loss expression of dbt in α, β and γ lobes of mushroom body (201Y) only during adulthood. n (UAS-dicer/+; UAS-dbtRNAi/+) = 52, n (201Y /+; tub-GAL80ts/+) = 41, n (UAS-dicer/201Y; UAS-dbtRNAi/tub-GAL80ts) = 40. (D) Total sleep of flies with loss expression of dbt in α, β and γ lobes of mushroom body (201Y) only during adulthood. n (UAS-dicer/+; UAS-dbtRNAi/+) = 52, n (201Y /+; tub-GAL80ts/+) = 41, n (UAS-dicer/201Y; UAS-dbtRNAi/tub-GAL80ts) = 40. (E) Sleep profile of flies with loss expression of dbt in α and β lobes of mushroom body (C309) only during adulthood. n (UAS-dicer/+; UAS-dbtRNAi/+) = 52, n (C309 /+; tub-GAL80ts/+) = 45, n (UAS-dicer/C309; UAS-dbtRNAi/tub-GAL80ts) = 36. (F) Total sleep of flies with loss expression of dbt in α and β lobes of mushroom body (C309) only during adulthood. n (UAS-dicer/+; UAS-dbtRNAi/+) = 52, n (C309 /+; tub-GAL80ts/+) = 45, n (UAS-dicer/C309; UAS-dbtRNAi/tub-GAL80ts) = 36. (G) Sleep profile of flies with loss expression of dbt in fan-shaped body (C205) only during adulthood. n (UAS-dicer/+; UAS-dbtRNAi/+) = 52, n (tub-GAL80ts/+; C205 /+) = 45, n (UAS-dicer/ tub-GAL80ts; UAS-dbtRNAi/C205) = 34. (H) Total sleep of flies with loss expression of dbt in fan-shaped body (C205) only during adulthood. n (UAS [file pgen.1010035.s007.tif]
